# Electronic Structure and Donor Ability of an Unsaturated Triphosphorus-Bridged Dimolybdenum Complex

**DOI:** 10.1021/acs.inorgchem.1c01552

**Published:** 2021-07-19

**Authors:** M. Angeles Alvarez, Melodie Casado-Ruano, M. Esther García, Daniel García-Vivó, Ana M. Guerra, Miguel A. Ruiz

**Affiliations:** Departamento de Química Orgánica e Inorgánica/IUQOEM, Universidad de Oviedo, E-33071 Oviedo, Spain

## Abstract

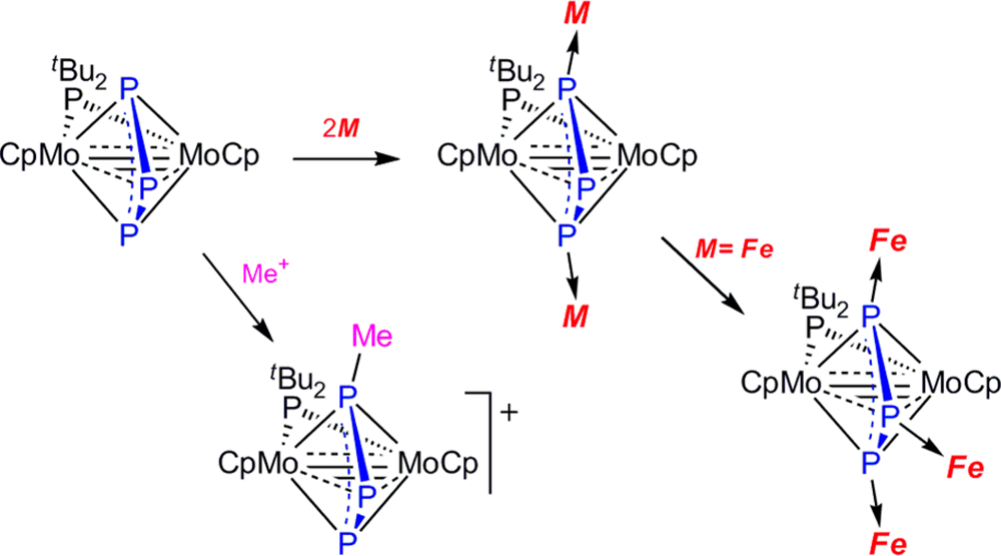

The triphosphorus complex [Mo_2_Cp_2_(μ-η^3^:η^3^-P_3_)(μ-P^*t*^Bu_2_)] was prepared in 83% yield by reacting the methyl complex [Mo_2_Cp_2_(μ-κ^1^:η^2^-CH_3_)(μ-P^*t*^Bu_2_)(μ-CO)] with P_4_ at 333 K, a process also giving small amounts of the methyldiphosphenyl complex [Mo_2_Cp_2_(μ-η^2^:η^2^-P_2_Me)(μ-P^*t*^Bu_2_)(CO)_2_]. The latter could be better prepared by first reacting the anionic complex Na[Mo_2_Cp_2_(μ-P^*t*^Bu_2_)(μ-CO)_2_] with P_4_ to give the diphosphorus derivative Na[Mo_2_Cp_2_(μ-η^2^:η^2^-P_2_)(μ-P^*t*^Bu_2_)(CO)_2_] and further reaction of the latter with MeI. Density functional theory calculations on the title complex revealed that its triphosphorus group can be viewed as an allylic-like P_3_^–^ ligand acting as a six-electron donor via the external P atoms, while coordination of the internal P atom involves donation from the π orbital of the ligand and back-donation to its π* orbital, both interactions having a weakening effect on the Mo–Mo and P–P connections. The reactivity of the title compound is dominated by the electron-donor ability associated with the lone pairs located at the P atoms. Its reaction with CF_3_SO_3_Me gave [Mo_2_Cp_2_(μ-η^3^:η^3^-P_3_Me)(μ-P^*t*^Bu_2_)](CF_3_SO_3_) as a result of methylation at an external atom of the P_3_ ligand, while its reaction with [Fe_2_(CO)_9_] enabled the addition of one, two, or three Fe(CO)_4_ fragments at these P atoms, but only the diiron derivative [Mo_2_Fe_2_Cp_2_(μ-η^3^:η^3^:κ^1^:κ^1^-P_3_)(μ-P^*t*^Bu_2_)(CO)_8_] could be isolated. This complex bears a Fe(CO)_4_ fragment at each of the external atoms of the P_3_ ligand, and the central P atom of the latter displays the lowest ^31^P chemical shift reported to date (δ_P_ −721.8 ppm). The related complexes [Mo_2_M_2_Cp_2_(μ-η^3^:η^3^:κ^1^:κ^1^-P_3_)(μ-P^*t*^Bu_2_)(CO)_10_] (M = Mo, W) were prepared by reacting the title compound with the corresponding [M(CO)_5_(THF)] complexes in toluene, while reaction with [Mo(CO)_4_(THF)_2_] also enabled the formation of the heptanuclear derivative [Mo_7_Cp_4_(μ-η^3^:η^3^:κ^1^:κ^1^-P_3_)_2_(μ-P^*t*^Bu_2_)_2_(CO)_14_]. The interatomic distances in the above compounds indicate that the central Mo_2_P_3_ skeleton of these molecules is little modified by the attachment of 16-electron M(CO)_*n*_ fragments at the external atoms of the P_3_ ligand.

## Abstract 2

In spite of the electronic unsaturation of the molecule, the reactivity of the title compound is dominated by the donor properties associated with the lone pairs of the allylic-like triphosphorus ligand, which can be easily methylated and can add up to three 16-electron metal carbonyl fragments M (M = Fe(CO)_4_, Mo(CO)_5_, and W(CO)_5_).

## Introduction

White phosphorus, an air-sensitive solid made up of tetrahedral P_4_ molecules, is the most important allotrope of phosphorus because most P-containing products manufactured today at the industrial scale, with the exception of fertilizers, are made ultimately from it. The production of all these molecular derivatives usually relies on intermediates and reagents environmentally unfriendly (e.g., chlorine to prepare PCl_3_ or PCl_5_), and this is why there is much current interest in finding ways to activate and functionalize the P_4_ molecule to yield useful derivatives, particularly organophosphorus compounds, by using more benign procedures. The main approaches to achieve this goal involve the use of either main-group reagents^1^ or suitable transition-metal complexes.^2^ Reactions of the latter complexes with P_4_ may yield derivatives containing a plethora of P_*n*_ ligands (*n* = 1–24), often displaying fascinating structures and unusual reactivity.^2,3^ However, these reactions many times require the use of strong thermal or photochemical activation to degrade the P_4_ molecule, then achieving this target with poor selectivity.

**Chart 1:**
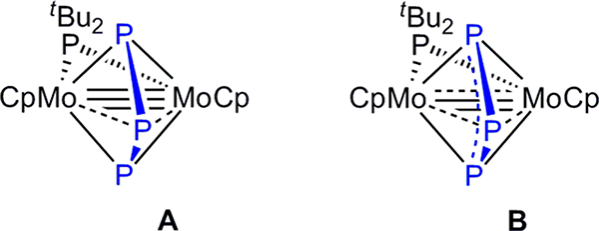
Different Descriptions of Bonding in Complex **2**

Recently, we found that the unsaturated methyl-bridged complex [Mo_2_Cp_2_(μ-κ^1^:η^2^-CH_3_)(μ-P^*t*^Bu_2_)(μ-CO)] (**1**) reacted selectively with P_4_ under relatively mild conditions (333 K) to give the triphosphorus-bridged derivative [Mo_2_Cp_2_(μ-η^3^:η^3^-P_3_)(μ-P^*t*^Bu_2_)] (**2**) in good yield, in a process formally involving the elimination of methylphosphinidene (PMe).^4^ There are two main points of interest concerning this compound: in the first place, we note that **2** is a rare example of a complex bearing a noncyclic P_3_ ligand bridging a dimetal center in a symmetrical η^3^:η^3^ mode. The only previously reported complexes of this type are the phosphorus sulfide complexes [Mo_2_Cp′_2_(μ-η^3^:η^3^-P_3_)(μ_2_-PS)] (Cp′ = C_5_Me_5_,^5^ C_5_H_2_^*t*^Bu_3_),^6^ the diiron radical [Fe_2_(C_5_H_2_^*t*^Bu_3_)_2_(μ-η^3^:η^3^-P_3_)], a product obtained in 2% yield from the reaction of [Fe_2_(C_5_H_2_^*t*^Bu_3_)_2_(CO)_4_(μ-κ^1^:κ^1^-P_4_)] with P≡C^*t*^Bu in refluxing toluene,^7^ and its anionic derivative [Fe_2_(C_5_H_2_^*t*^Bu_3_)_2_(μ-η^3^:η^3^-P_3_)]^−^, a complex recently prepared by degrading the above P_4_-bridged complex with a NHC ligand and thought to contain no metal–metal bond.^8^ As a result of all of the above, only very limited chemistry of the acyclic P_3_ ligand in such a coordination mode has been explored to date, it being restricted to some reactions of the mentioned dimolybdenum complexes with [Cr(CO)_5_(THF)]^5^ and with different M(I)-based electrophiles (M = Cu, Ag).^6,9^ On the other hand, a description of the chemical bonding in **2** was not obvious itself because application of the 18-electron rule to this molecule, if considering the P^*t*^Bu_2_ and P_3_ ligands as three- and five-electron donors, respectively (neutral counting scheme), would lead to the formulation of a Mo–Mo triple bond for this molecule (**A** in Chart 1). However, this was rather inconsistent with the actual intermetallic separation of 2.6221(3) Å in **2**, significantly longer than expected for a 30-electron complex with P-bridging ligands (cf. ca. 2.51 Å in [Mo_2_Cp_2_(μ-PPh_2_)_2_(μ-CO)]).^10^ On the basis of these considerations, we decided to analyze in more detail the electronic structure of this unusual complex by using density functional theory (DFT) methods, while also broadly exploring its chemical behavior by reacting it with some p-block molecules and different transition-metal carbonyl complexes, all of which is the subject of the present paper. The latter reactions were of particular interest because previous work from our lab has proven that addition of M(CO)_*n*_ fragments could ultimately induce P–P bond cleavage processes on the related η^2^:η^2^-bridged diphosphenyl complex [Mo_2_Cp_2_(μ-η^2^:η^2^-P_2_Me)(μ-PCy_2_)(CO)_2_],^11^ although not on its anionic diphosphorus-bridged precursor [Mo_2_Cp_2_(μ-η^2^:η^2^-P_2_)(μ-PCy_2_)(CO)_2_]^−^.^12^ As discussed below, our calculations on **2** indicate that its triphosphorus ligand can be viewed as an allylic-like P_3_^–^ anion contributing with six electrons to the dimetal center via the external P atoms, while coordination of the internal P atom has a weakening effect on both the Mo–Mo and P–P connections. As a result, the intermetallic and P–P bond orders become lower than 3 and 1.5, respectively (**B** in Chart 1). In spite of the electronic unsaturation of the molecule, the chemical behavior of **2** is dominated by the electron-donor ability associated with the lone pairs located at the P atoms of the triphosphorus ligand, which can actually bind up to three metal–carbonyl fragments.

## Results and Discussion

### Synthesis and Molecular Structure of the Triphosphorus Complex **2**

**Scheme 1:**
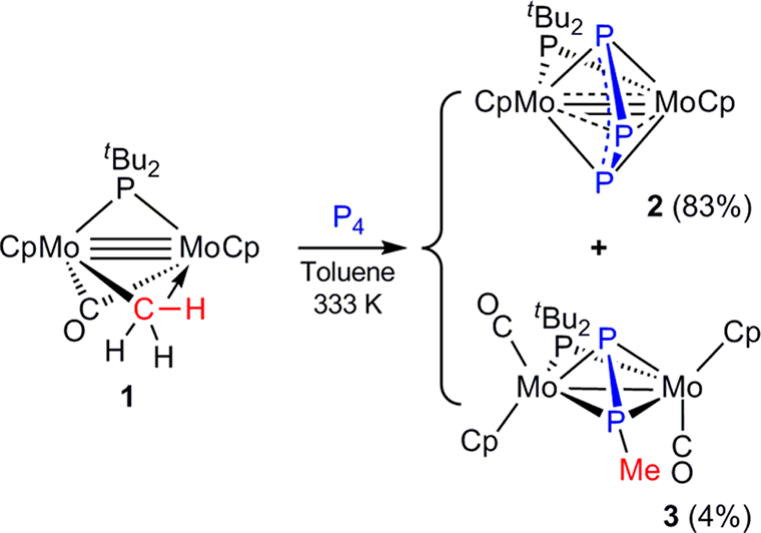
Preparation of Compound **2**

Compound **1** reacts with stoichiometric amounts of white phosphorus under a gentle heating (333 K) in toluene solution to give the dark blue triphosphorus-bridged complex [Mo_2_Cp_2_(μ-η^3^:η^3^-P_3_)(μ-P^*t*^Bu_2_)] (**2**) as a major product (83% yield after chromatographic work-up) along with small amounts (ca. 5%) of the methyldiphosphenyl complex [Mo_2_Cp_2_(μ-η^2^:η^2^-P_2_Me)(μ-P^*t*^Bu_2_)(CO)_2_] (**3**) (Scheme 1).

**Chart 2:**
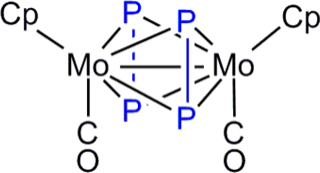
Structure of [Mo_2_Cp_2_(μ-η^2^:η^2^-P_2_)_2_(CO)_2_]

The formation of **2** formally results from elimination of methylphosphinidene (“PMe”) between **1** and P_4_, a rare process itself for which we cannot quote a precedent, and decarbonylation; unfortunately, NMR analysis of the crude reaction mixture did not enable us to determine the fate of this unstable phosphinidene. As for the formation of **3**, first unnoticed in our preliminary study of this reaction,^4^ one might be tempted to think it as derived from reaction of white phosphorus with the dicarbonyl complex [Mo_2_Cp_2_(μ-κ^1^:η^2^-CH_3_)(μ-P^*t*^Bu_2_)(CO)_2_], which is the actual precursor of compound **1**,^13^ therefore a potential contaminant of the starting material in this reaction. However, separated experiments revealed that the above dicarbonyl complex does not react with P_4_ at 333 K. It did it, however, in toluene solution at ca. 405 K, but then no detectable amounts of **3** were formed either. Instead, a mixture of the new diphosphorus complex [Mo_2_Cp_2_(μ-η^2^:η^2^-P_2_)_2_(CO)_2_] (Chart 2)^14,15^ and other yet uncharacterized products was formed. Thus, it is concluded that **3** is formed genuinely from **1** and P_4_, even if through a minor reaction pathway also involving reaction with part of the carbon monoxide released in the formation of the main product **2**. A more specific method to prepare the diphosphenyl complex **3** is discussed below.

**Figure 1:**
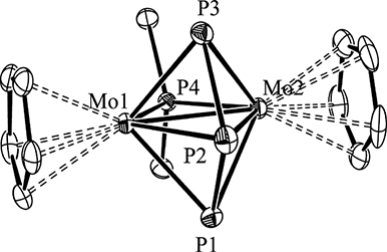
ORTEP diagram (30% probability) of compound **2**, with ^*t*^Bu (except their C^1^ atoms) and H atoms omitted for clarity.^4^ Selected bond lengths (Å) and angles (deg): Mo1–Mo2 = 2.6221(3); Mo1–P1 = 2.3952(8); Mo2–P1 = 2.3952(8); Mo1–P2 = 2.6308(8); Mo2–P2 = 2.6255(8); Mo1–P3 = 2.3988(8); Mo2–P3 = 2.3909(8); Mo1–P4 = 2.4224(7); Mo2–P4 = 2.4131(7); P1–P2 = 2.1556(12); P2–P3 = 2.1442(12). P1–Mo1–P4 = 91.18(3); P2–Mo1–P4 = 116.91(3); P3–Mo1–P4 = 94.86(3); P1–P2–P3 = 107.47(4).

The structure of **2** in the crystal (Figure 1) was determined during our preliminary study of the reactivity of the methyl complex **1**.^4^ The acyclic P_3_ ligand bridges symmetrically the dimetal center, with the external P atoms being tightly bound to the Mo atoms, as judged from the short Mo–P distances of ca. 2.40 Å, actually a bit shorter than the Mo–P distances involving the P^*t*^Bu_2_ ligand (ca. 2.41 Å). In contrast, the interaction of the internal P atom with the metal atoms is much weaker, as expected (Mo–P ca. 2.63 Å). The P–P bond lengths of ca. 2.15 Å in **2** are shorter than the interatomic separation in the P_4_ molecule (2.21 Å), which is indicative of the presence of some multiplicity in these bonds, a matter to be discussed below, and also are shorter than those recently measured in the anionic complex [Fe_2_(C_5_H_2_^*t*^Bu_3_)_2_(μ-η^3^:η^3^-P_3_)]^−^ (2.1601(8) and 2.1897(8) Å).^8^ As noted above, the intermetallic separation of 2.6221(3) Å in **2** is longer than expected for a Mo–Mo triple bond. However, it is still shorter than the distances found for Mo–Mo double bonds in related species bearing two P-donor bridging ligands (cf. 2.71 Å in [Mo_2_Cp_2_(μ-PPh_2_)_2_(CO)_2_]^10^ or 2.749(2) Å for [Mo_2_WCp_2_(μ_3_-P)(μ-PCy_2_)(CO)_7_],^11a^ a matter also to be discussed below.

Spectroscopic data in solution for **2** (Table 1 and Experimental Section) are consistent with the symmetrical structure found in the crystal. In particular, we note that the ^1^H and ^13^C{^1^H} NMR spectra display single resonances for the Cp and ^*t*^Bu groups, which remained unchanged down to 193 K. In addition, the ^31^P spectrum displays a single and strongly deshielded doublet resonance for the external atoms of the P_3_ chain (δ_P_ 412 ppm, *J*_PP_ = 405 Hz), whereas the central P atom gives rise to an extremely deshielded triplet resonance at −626.5 ppm. The large P–P coupling in **2** exceeds the usual values of ca. 160–370 Hz found in diphosphines having conventional alkyl or aryl substituents^16^ and approaches the figures of 510–670 Hz found in diphosphenes,^17^ this being again indicative of multiplicity in the P–P bonding at the P_3_ chain. Besides this, we should remark that the chemical shift for the central P atom in **2** is far lower (by some 250 ppm) than the ones previously determined for the few η^3^:η^3^-bridged complexes reported to date (ca. −375 ppm in [Mo_2_Cp′_2_(μ-η^3^:η^3^-P_3_)(μ_2_-PS)]^5,6^ and ca. −380 ppm in [Fe_2_(C_5_H_2_^*t*^Bu_3_)_2_(μ-η^3^:η^3^-P_3_)]^*n*^ (*n* = 0, −1)).^7,8^ Finally, we note that the ^31^P chemical shift of the P^*t*^Bu_2_ ligand in **2** (δ_P_ 176.1 ppm) is significantly lower than those typically found for related complexes with Mo–Mo triple bonds (cf. 266.2 ppm in **1**)^13^ but is actually similar to those found for complexes of type *trans*-[Mo_2_Cp_2_(μ-P^*t*^Bu_2_)(μ-PRR′)(CO)_2_] (ca. 172 ppm),^18^ for which a Mo–Mo double bond is to be proposed according to the 18-electron rule. All of this in agreement with the results of DFT calculations discussed below.

**Table 1:**
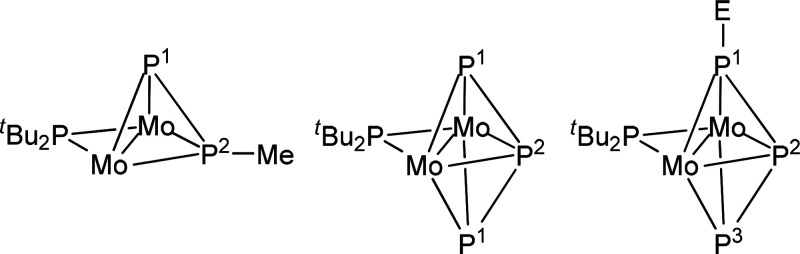
Selected ^31^P{^1^H} NMR Data for New Compounds^a^

compound	δ (P^*t*^Bu_2_)	δ (P^1^)	δ (P^2^)	δ (P^3^)	*J*(P^1^–P^2^)	*J*(P^2^–P^3^)
[Mo_2_Cp_2_(μ-η^3^:η^3^-P_3_)(μ-P^*t*^Bu_2_)] (**2**)^b^	176.1	412.0	–626.5		405	
[Mo_2_Cp_2_(μ-η^2^:η^2^-P_2_Me)(μ-P^*t*^Bu_2_)(CO)_2_] (**3**)	223.5	–286.0	–84.9		524	
Na[Mo_2_Cp_2_(μ-η^2^:η^2^-P_2_)(μ-P^*t*^Bu_2_)(CO)_2_] (**5**)^c^	222.9	–156.9^c^				
[Mo_2_Cp_2_(μ-PH_2_)(μ-P^*t*^Bu_2_)(CO)_2_] (**6**)	175.8		–55.5^d^			
[Mo_2_Cp_2_(μ-η^3^:η^3^-P_3_Me)(μ-P^*t*^Bu_2_)](CF_3_SO_3_) (**7**)^e^	188.7	315.4	–699.5	444.3	410	423
[Mo_2_FeCp_2_(μ-η^3^:η^3^:κ^1^-P_3_)(μ-P^*t*^Bu_2_)(CO)_4_] (**8**)	179.8	377.6^f^	–648.5	455.2^f^	420	405
[Mo_2_Fe_2_Cp_2_(μ-η^3^:η^3^:κ^1^:κ^1^-P_3_)(μ-P^*t*^Bu_2_)(CO)_8_] (**9**-**Fe**)	185.7	417.8	–721.8		422	
[Mo_4_Cp_2_(μ-η^3^:η^3^:κ^1^:κ^1^-P_3_)(μ-P^*t*^Bu_2_)(CO)_10_] (**9**-**Mo**)^e^	184.2	391.6	–687.3		394	
[Mo_2_W_2_Cp_2_(μ-η^3^:η^3^:κ^1^:κ^1^-P_3_)(μ-P^*t*^Bu_2_)(CO)_10_] (**9**-**W**)	185.2	354.3	–689.8		391	
[Mo_2_Fe_3_Cp_2_(μ-η^3^:η^3^:κ^1^:κ^1^:κ^1^-P_3_)(μ-P^*t*^Bu_2_)(CO)_12_] (**10**)^g^	177.0	404.3	–552.0		445	
[Mo_7_Cp_4_(μ-η^3^:η^3^:κ^1^:κ^1^-P_3_)_2_(μ-P^*t*^Bu_2_)_2_(CO)_14_] (**11**)	183.7	383.8^f^	–678.6	403.8^f^	380	414

a NMR data recorded in C_6_D_6_ solution at 121.48 MHz and 293 K, with chemical shifts (δ) in ppm relative to external 85% aqueous H_3_PO_4_, and P–P couplings (*J*_PP_) in hertz. Labels according to the figure shown above (E = electrophile).

b Data taken from ref (4).

c In tetrahydrofuran solution; averaged resonance for P^1^ and P^2^ atoms; see text.

d Resonance for the PH_2_ ligand.

e In CD_2_Cl_2_ solution.

f Assignment of the P^1^ and P^3^ resonances might be exchanged; see text.

g In toluene solution.

### Electronic Structure of Compound **2**

To better understand the geometry and chemical behavior of compound **2**, we analyzed its geometric and electronic structure using DFT methods (see the Experimental Section and the Supporting Information).^19^ First we note that the optimized structure for **2** was in excellent agreement with the one determined in the crystal, with the P_3_ ligand symmetrically bridging the dimetal center and displaying P–P distances (ca. 2.17 Å) shorter than expected for single bonds, while the value of the Mo–Mo separation (2.633 Å) can be considered intermediate between the figures expected for triple and double bonds, as discussed above (Table 2).

**Table 2:** M06L-DFT Computed Bond Lengths (Å) and Angles (deg) for **2** along with Some Topological Properties of the Electron Density at the Corresponding Bond Critical Points^a^

	distance/angle	expt	ρ	∇^2^ρ
Mo–Mo	2.633	2.6221(3)	0.516	1.262
Mo–P^*t*^Bu	2.424	2.418(1)	0.554	2.948
Mo–P1	2.413	2.397(1)	0.573	2.245
Mo–P2	2.654	2.632(1)	0.378	2.374
P1–P2	2.167	2.150(1)	0.725	–1.911
P1–P2–P3	107.35	107.47(4)		

a Average values for the nearly equivalent bonds of each type, with labeling scheme as for Table 1. Values of the electron density at the bond critical points (ρ) are given in e Å^–3^; values of the Laplacian of ρ at these points (∇^2^ρ) are given in e Å^–5^.

**Figure 2:**
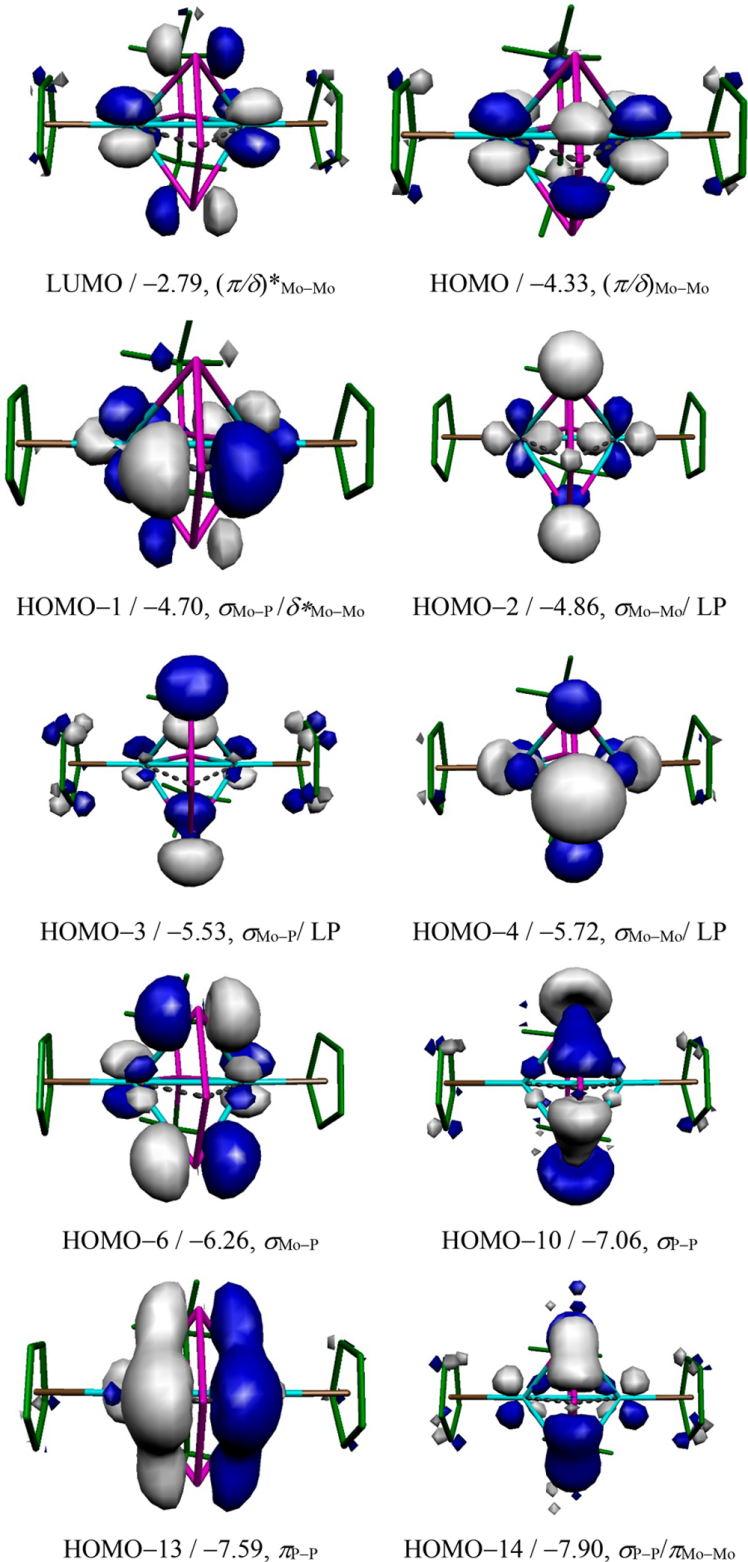
Selected M06L-DFT computed molecular orbitals of compound **2** viewed from a point close to the Mo–P(^*t*^Bu_2_)–Mo plane, with their energies (in eV) and main bonding character indicated below (LP stands for lone pair character at the P atoms). A view of these orbitals from a plane perpendicular to the above one can be found in the Supporting Information.

The frontier Kohn–Sham molecular orbitals computed for **2** (Figure 2) show an extensive mixing of Mo–P and Mo–Mo bonding as well as mixing with the P-based nonbonding orbitals representing the expected lone electron pairs at the P atoms. The latter can be recognized particularly in the HOMO–2, −3, and −4 orbitals, the first of them and the latter one also having σ(Mo–Mo) bonding character. The intermetallic bonding is completed with a hybrid π/δ bonding interaction, actually the HOMO of the molecule, of which the LUMO is the corresponding antibonding combination. Other intermetallic interactions can be recognized in the HOMO–14 orbital (π bonding character), HOMO–13 (σ* character), and HOMO–1 (δ* character), so the intermetallic bond order should be lower than 3. It is interesting to analyze the interactions of the phosphorus orbitals perpendicular to the P_3_ plane. The classical combinations expected for an angular P_3_ unit (ozone- or allylic-like) involves bonding, nonbonding, and antibonding combinations.^20−22^ These can be recognized in orbitals HOMO–13, −6, and −1, respectively. The HOMO–13 has positive overlaps with acceptor orbitals of the metals in the Mo_2_P^*t*^Bu_2_ plane and represents a bonding interaction of the central P atom of the P_3_ ligand with the metal atoms, with some σ*(Mo–Mo) character, as noted above. On the other hand, the HOMO–1 involves the π(P–P) antibonding combination of the P_3_ unit, which is empty in the free P_3_^–^ ligand; accordingly, this orbital, which also has δ*(Mo–Mo) character, can be viewed as representing a back-donation from the dimetal center to the P_3_ ligand. These two orbitals would then account for the bonding between the dimetal center and the internal P atom of the triphosphorus chain, but have a weakening effect on the P–P bonds (also on the Mo–Mo bond). In agreement with this, the experimental P–P distance in **2** of ca. 2.15 Å is significantly lower than the one in white phosphorus (2.21 Å), but still far from the reference length of 2.05 Å for P–P double bonds.^17,23^ Finally, the HOMO–6 orbital represents a bonding interaction between the external atoms of the P_3_ chain and the dimetal center. This orbital follows from interaction of metal orbitals with the nonbonding π orbital of the P_3_^–^ ligand, which is at first surprising since the latter is expected to be empty in the free P_3_^–^ ligand, if we assume that the terminal P atoms bear two lone electron pairs each and the central P atom just one. However, this is not the case. According to a DFT calculation at the same level as the one used for **2**, a P_3_^–^ ion with an imposed P–P–P angle identical with the one determined for **2** (107.35°, *d*_PP_ = 2.062 Å) has a configuration of type (σ_1_)^2^(σ_2_)^2^(σ_3_*)^2^(σ_4_)^2^(σ_5_)^2^(π)^2^(π^nb^)^2^(σ_6_*)^2^ (see the Supporting Information). The population of the π^nb^ orbital is somewhat unexpected on simple electron counting (allocating five lone pairs at the P atoms would leave only two electrons for the π manifold) and perhaps is favored to reduce repulsions with other nonbonding electron pairs. In any case, this filled π^nb^ orbital has the right angular distribution to interact efficiently with acceptor orbitals of the dimetal center as in the HOMO–6 and ultimately enables the P_3_^–^ ligand to act as a six-electron donor via the external P atoms, in agreement with the short Mo–P distances observed, which are comparable to the ones involving the P^*t*^Bu_2_ ligand, as noted above.

Analysis of the electron densities at the different bond critical points (bcp) of **2** under the atoms in molecules (AIM)^24^ scheme (Table 2) renders a picture essentially consistent with the above MO analysis but additionally gives a more precise picture of the result of mutually canceling bonding and antibonding interactions operating in the molecule. The electron density at the intermetallic bcp (0.516 e Å^–3^) has a value intermediate between the figures previously computed by us at a similar level for related dimolybdenum species bearing Mo–Mo triple (ca. 0.60 e Å^–3^)^25^ and double bonds (0.43 e Å^–3^),^26^ and the electron densities at the Mo–P bcp’s of the external atoms of the P_3_ ligand (ca. 0.57 e Å^–3^) are comparable to those involving the P^*t*^Bu_2_ ligand. As expected, the latter figures nearly double the values for the connections between the central P atom and the molybdenum atoms (0.378 e Å^–3^). As for the P–P bonding in **2**, we note that the electron densities of ca. 0.725 e Å^–3^ at the corresponding bcp’s are lower than the value of 0.912 e Å^–3^ computed for the free P_3_^–^ anion under imposed geometry (P–P–P = 107.35°) and approach the electron densities computed at the same level for the bcp’s of P_4_ (0.715 e Å^–3^, see the Supporting Information).^27^ This suggests that the π(P–P) bonding interaction in complex **2** represented by the HOMO–13 is canceled to a significant extent by the antibonding interaction implied by the HOMO–1.

In summary, on the basis of the above MO and AIM analysis, we conclude that the triphosphorus ligand in **2** can be viewed as an allylic-like P_3_^–^ ligand acting as a six-electron donor via the external P atoms, while the binding of the central P atom involves donation from the π orbital of the P_3_^–^ ligand to the dimetal center, and back-donation of the latter to the π* orbital of the P_3_^–^ ligand, these orbital interactions having a weakening effect on both the Mo–Mo and P–P connections. As a result of all of this, the intermetallic bond in **2** displays geometric and topological properties intermediate between those of double and triple bonds, while the properties of the P–P bond are intermediate between those of single and allylic-like (bond order 1.5) interactions. We have tried to illustrate this intermediate bonding situation in **2** by using the chemical diagram **B** of Chart 1.

### Preparation of the Diphosphenyl Complex **3**

**Scheme 2:**
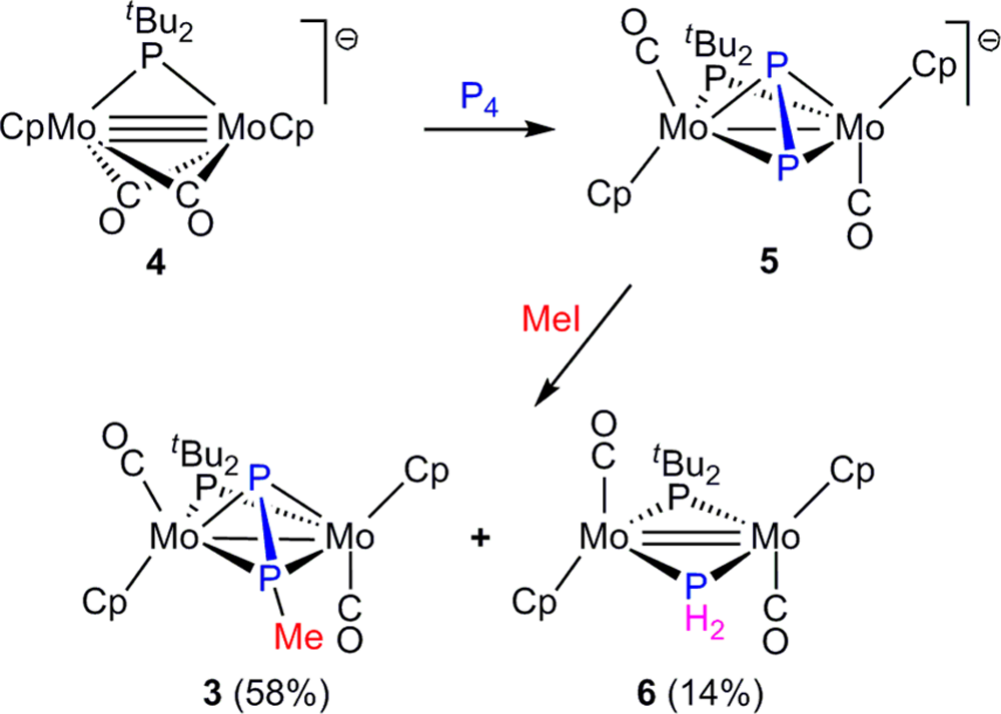
Preparation of Compound **3**

To prepare complex **3** in significant amounts, we followed the route previously developed by us to synthesize related PCy_2_-bridged dimolybdemun^28^ and ditungsten complexes.^29^ This starts with the room temperature reaction of the unsaturated anion [Mo_2_Cp_2_(μ-P^*t*^Bu_2_)(μ-CO)_2_]^−^ (**4**) (Na^+^ salt) with white phosphorus to give the Na^+^ salt of the diphosphorus-bridged complex [Mo_2_Cp_2_(μ-η^2^:η^2^-P_2_)(μ-P^*t*^Bu_2_)(CO)_2_]^−^ (**5**) almost quantitatively (Scheme 2). In a second step, the latter complex is reacted with methyl iodide at 273 K to give the desired diphosphenyl-bridged complex **3**, which can be isolated in ca. 60% yield upon chromatographic work-up. In this reaction, however, significant amounts of the PH_2_-bridged complex [Mo_2_Cp_2_(μ-PH_2_)(μ-P^*t*^Bu_2_)(CO)_2_] (**6**) were also formed, likely resulting from a side hydrolytic process, not investigated. We note that Mays and co-workers have shown previously that reacting the neutral diphosphorus-bridged complexes [M_2_Cp_2_(μ-η^2^:η^2^-P_2_)(CO)_4_] with M′OH (M = Mo; W: M′ = Na, K), in tetrahydrofuran–H_2_O (400/1) at 353 K, yields the corresponding PH_2_-bridged anions [M_2_Cp_2_(μ-PH_2_)(CO)_4_]^−^ in good yield.^30^ The presence of water is clearly critical in this P_2_ to PH_2_ conversion, since recent work by Scheer and co-workers has shown that reaction of the above dimolybdenum complex with KOH in pure tetrahydrofuran is very slow, it only being completed after 7 days in tetrahydrofuran solution at 333 K.^31^

Spectroscopic data for **5** are comparable to those of its PCy_2_-bridged Mo and W analogues^28,29^ and only deserve a few comments. In particular, its IR spectrum displays three rather than two C–O stretches, at 1833 (vs), 1758 (w), and 1695 (s) cm^–1^, which is indicative of the presence of more than one species in solution. The most prominent bands are assigned to a tight ion pair involving the Na^+^ cation and the O atom of one of the carbonyl ligands of the anion, while the weak band at 1758 cm^–1^ is assigned to the asymmetric C–O stretch of the solvent-separated anion; the symmetric C–O stretch expected for this minor species would be obscured by the strong 1883 cm^–1^ band of the dominant ion pair. Both species interconvert in solution rapidly on the NMR time scale, as the ^31^P NMR spectrum of **5** at room temperature displays single resonances for both the P^*t*^Bu_2_ and P_2_ ligands, at 222.9 and −156.9 ppm, respectively. The latter is in turn an average resonance of the two resonances expected for the inequivalent P atoms of the diphosphorus ligand in this fluxional complex, as shown by theoretical and experimental work on the PCy_2_-bridged Mo_2_ analogue of **5**. Indeed, the Li^+^ salt of the latter complex gives an averaged P_2_ resonance at −176.5 ppm at room temperature, which splits into resonances at −90.0 and −273.0 ppm upon cooling.^28^

Spectroscopic data for **3** (Table 1 and Experimental Section) also are comparable to those of its PCy_2_-bridged Mo and W analogues, with only a few significant differences. In particular, we note that its IR spectrum displays two C–O stretches at 1884 (s) and 1801 (vs) cm^–1^. The symmetric stretch here is more intense than in the case of the PCy_2_-bridged analogues (there being of just medium intensity), which denotes a stronger deviation of the CO ligands from an ideal antiparallel arrangement.^32^ This is a structural difference that we attribute to the steric effect of the bulky P^*t*^Bu_2_ ligand, which would promote a larger puckering of the central Mo_2_PX skeleton of the molecule (compared to the PCy_2_-bridged complex), whereby one carbonyl ligand would point further away from the dimetal unit (Mo–Mo–CO > 90°) while the other one would lean to the intermetallic bond (Mo–Mo–CO < 90°). As a result, the angle defined by the CO ligands would be lower than in the PCy_2_-bridged complex (144.4°).^28^ This sort of geometrical distortion has been previously observed by us in other dicarbonyl complexes of the type [M_2_Cp_2_(μ-PCy_2_)(μ-X)(CO)_2_] bearing space-demanding bridging groups (M = Mo, W; X = SnPh_3_, HCN^*t*^Bu, SCPh, etc.).^33^

As for the NMR parameters of **3**, we note that the strong coupling between the inequivalent phosphorus atoms of the diphosphenyl ligand (δ_P_ −84.9 and −286.0 ppm, *J* = 524 Hz), close to the values measured for free diphosphenes (510–670 Hz),^17^ is therefore indicative of retention of substantial multiplicity in that bond, a matter already analyzed by us for the PCy_2_ analogue.^28^ Finally, we note that each of the inequivalent carbonyl ligands displays one quite large (40/28 Hz) and two small P–C couplings. This is consistent with the structure determined for the PCy_2_-bridged analogue of **3** and the known dependence of two-bond P–M–C couplings with the corresponding angle in this type of complex,^34^ whereby the largest carbonyl couplings in **3** can be assigned to couplings with the apical P atom of the diphosphenyl ligand, as these involve the most extreme angles (ca. 67° and 131° in the PCy_2_ complex).

Spectroscopic data for **6** are comparable to those of the large family of mixed-phosphanyl complexes of type *trans*-[M_2_Cp_2_(μ-PR_2_)(μ-PR′R″)(CO)_2_] (M = Mo, W) previously prepared by us^18^ and then deserve only a few comments. We just note that the bridging PH_2_ group gives rise to a diagnostic highly shielded ^31^P NMR resonance (δ_P_ −55.5 ppm) strongly coupled to two equivalent H atoms (δ_H_ 4.71 ppm; *J*_PH_ = 363 Hz) and very weakly coupled to the P atom of the P^*t*^Bu_2_ group (*J*_PP_ = 7 Hz). The latter is a persistent spectroscopic feature found for all these *trans*-dicarbonyl complexes displaying an essentially flat Mo_2_P_2_ central core. Another characteristic feature of these complexes, for which a metal–metal double bond is to be formulated according to the 18-electron rule and DFT calculations,^26^ is the relatively poor deshielding of their P atoms (compared with PR_2_ ligands bridging single or triple bonds). Indeed, the ^31^P chemical shift of the P^*t*^Bu_2_ ligand in **6** (δ_P_ 175.8 ppm) compares well with the observed shift for the isoelectronic complex [Mo_2_Cp_2_(μ-P^*t*^Bu_2_)(μ-PPh_2_)(CO)_2_] (174.1 ppm)^18^ and is significantly lower than the chemical shifts of the P^*t*^Bu_2_ ligands in complexes [Mo_2_Cp_2_(μ-H)(μ-P^*t*^Bu_2_)(CO)_4_] (Mo–Mo single bond, δ_P_ 267.5 ppm)^35^ and [Mo_2_Cp_2_(μ-P^*t*^Bu_2_)(μ-PPh_2_)(μ-CO)] (Mo–Mo triple bond, δ_P_ 288.9 ppm).^18^ An analogous comment can be made on the PH_2_ resonance of **6** (δ_P_ −55.5 ppm), which appears some 90 ppm upfield from the one observed for the electron-precise complex [Mo_2_Cp_2_(μ-H)(μ-PH_2_)(CO)_4_] (δ_P_ +33.7 ppm).^30^

### Acid–Base Chemistry of Complex **2**. Methylation Reactions

By considering the electronic unsaturation of **2**, discussed above, and particularly the Mo–Mo antibonding nature of the LUMO of the molecule, we would expect it to easily add at the dimetal site simple donors such as CO or isocyanide ligands, so as to give electron-precise derivatives. However, such reactions do not take place under ordinary conditions, perhaps due to the steric shielding that the Cp and bridging ligands of **2** provide to the unsaturated dimetal center of the molecule. Reactions that aimed to check possible insertions into the P–P or Mo–P bonds of the triphosphorus ligand of **2** (activated alkynes such as RC≡CCO_2_Me, with R = H, CO_2_Me) also failed to occur even in refluxing toluene solution or under UV–vis irradiation. In fact, the chemistry of **2** seems to be dominated by the nucleophilic properties associated with the lone electron pairs at the P atoms of the triphosphorus ligand, as shown by its easy methylation, discussed below, and addition of 16-electron metal carbonyl fragments, to be discussed separately. Surprisingly, however, no reaction was observed between **2** and a prototypal Lewis acid such as borane (no reaction with BH_3_·THF in toluene solution).

**Chart 3:**
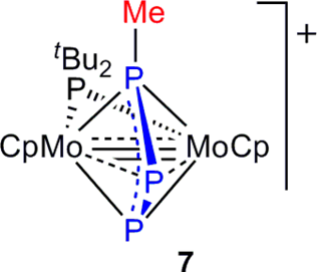
Structure of Compound **7**

Compound **2** does not react with MeI at room temperature. However, reaction of **2** with methyl triflate takes place rapidly at 253 K to give the salt [Mo_2_Cp_2_(μ-η^3^:η^3^-P_3_Me)(μ-P^*t*^Bu_2_)](CF_3_SO_3_) (**7**) in an almost quantitative way as a result of the incorporation of a methyl cation at one of the external P atoms of the triphosphorus ligand (Chart 3). Such a stereoselectivity is immediately deduced from the presence, in the ^31^P NMR spectrum of **7**, of three mutually coupled resonances corresponding to the former P_3_ ligand and from the fact that only one of them is significantly broadened upon switching off the ^1^H decoupler. This circumstance, along with the number of large one-bond P–P couplings (410 and 423 Hz), enable full assignment of the ^31^P resonances of **7** (Table 1).

**Figure 3:**
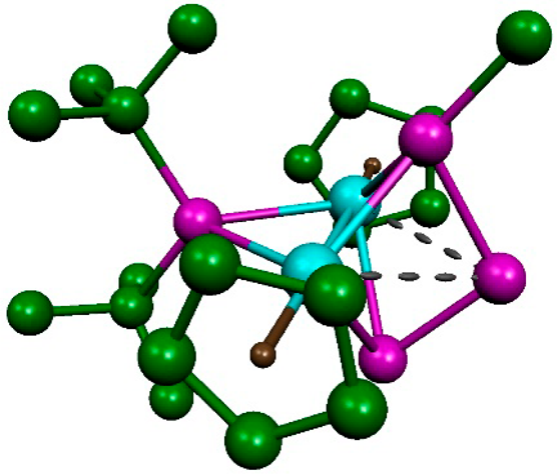
M06L-DFT optimized structure of the cation in compound **7**, with H atoms omitted for clarity. Selected bond lengths (Å, labels as in Table 1): Mo–Mo = 2.711; Mo–P1 = 2.343; Mo–P2 = 2.698; Mo–P3 = 2.431; Mo–P^*t*^Bu_2_ = 2.447; P1–P2 = 2.182; P2–P3 = 2.152.

Attachment of the Me^+^ cation at the external P atom of the P_3_ chain has little effect on the P–P couplings but implies a strong shielding for the P atom involved (by some 100 ppm) and the central P atom (by some 75 ppm), while the remaining external P atom is deshielded by 40 ppm. These shielding effects are different from those observed for the diphosphorus complex **5** and their PCy_2_ analogues, which upon methylation undergo a strong deshielding of some 200 ppm at the P atom involved and a shielding of some 200 ppm at the remaining P atom of the diphosphorus ligand. In addition, we note that the ^31^P chemical shift of the P^*t*^Bu_2_ ligand in **7** (188.7 pm) is comparable to the one in **2**, which suggests that the intermetallic interaction of **2** remains in the range of double bonds upon methylation. Actually, the structure computed for this cation (Figure 3) displays an intermetallic separation of 2.711 Å, longer than the one computed for **2** (2.633 Å). This lengthening effect can be understood by recalling that the frontier molecular orbital of **2** most likely involved in the formation of **7** (HOMO–2) has some σ(Mo–Mo) bonding character. Noticeably, the added Me group lies in the corresponding Mo_2_P plane, which renders a distorted trigonal-pyramidal geometry at the corresponding P atom, an effect previously observed upon alkylation of the PCy_2_ analogue of the diphosphorus complex **5**.^28^ We finally note that we also computed the structure of an isomer of the cation in **7** having the Me group bound to the central atom of the triphosphorus ligand (**7′**, see the Supporting Information). This P atom might be viewed as sterically more accessible for binding to an external electrophile. However, the computed Gibbs free energy for such an isomer was 54 kJ/mol higher than that of **7**, this suggesting that the observed site preference in the methylation of **2** has an electronic origin, not obvious to us at the moment.

### Addition of Fe(CO)_4_ Fragments to Complex **2**

**Scheme 3:**
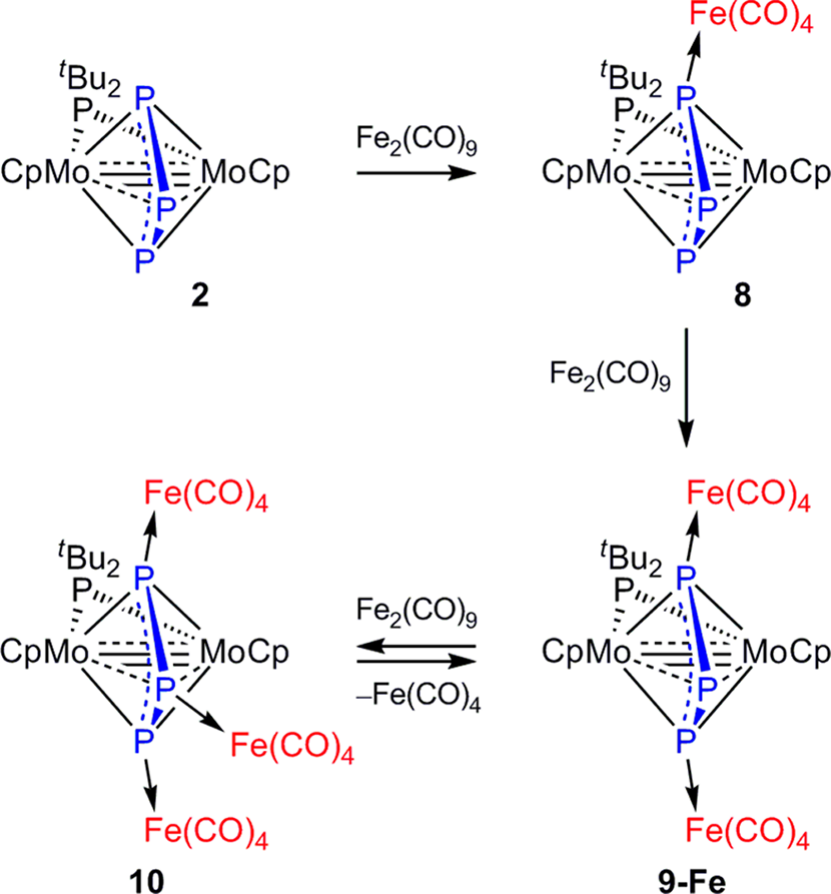
Iron Derivatives of Complex **2**

The reaction of **2** with [Fe_2_(CO)_9_], a well-stablished precursor of the 16-electron fragment Fe(CO)_4_, turned out to be quite sensitive to the relative amount of the diiron reagent used and in all cases proceeded rapidly in toluene solution at room temperature. When using stoichiometric or under-stoichiometric amounts of the diiron reagent, the major product was the iron derivative [Mo_2_FeCp_2_(μ-η^3^:η^3^:κ^1^-P_3_)(μ-P^*t*^Bu_2_)(CO)_4_] (**8**) along with smaller amounts of the diiron derivative [Mo_2_Fe_2_Cp_2_(μ-η^3^:η^3^:κ^1^:κ^1^-P_3_)(μ-P^*t*^Bu_2_)(CO)_8_] (**9-Fe**) (Scheme 3). However, the former could not be isolated, as it decomposed upon all attempts of isolation, to yield **2** and **9-Fe**. As expected, the diiron complex was the major product formed when reacting **2** with 2 equiv of [Fe_2_(CO)_9_]. In that case, however, a ^31^P spectrum of the crude reaction mixture revealed the presence of small amounts of another new species, identified as the triiron derivative [Mo_2_Fe_3_Cp_2_(μ-η^3^:η^3^:κ^1^:κ^1^:κ^1^-P_3_)(μ-P^*t*^Bu_2_)(CO)_12_] (**10**). Increasing the amount of [Fe_2_(CO)_9_] used in this reaction expectedly led to an increase in the relative amount of the triiron derivative present in the final reaction mixture. However, this pentanuclear complex could not be isolated either, as it progressively decomposed to give **9-Fe** as the only organometallic product.

The identification of compounds **8** and **10** is only based on the corresponding ^31^P NMR spectra and the conditions under which each of them is best formed, already noted above. Compound **8** displays a P^*t*^Bu_2_ resonance similar to that of **2** and three strongly coupled resonances for the P_3_ ligand at 455.2, 377.6, and −648.5 ppm (*J*_PP_ = 420 and 405 Hz, Table 1). These spectroscopic data are qualitatively similar to those of the methylation product **7** and are thus indicative of the attachment of a single Fe(CO)_4_ fragment to one of the external P atoms of the triphosphorus ligand. In this case we could not identify spectroscopically the resonance for the Fe-bound P atom, and we propose it to be the one being shielded with respect to the corresponding resonance in the parent compound (δ_P_ 377.6 ppm) by analogy with the changes observed in the formation of **7**. In the case of compound **10**, the assignment of P_3_ resonances is straightforward, as it displays a doublet resonance at 404.3 ppm (*J* = 445 Hz) for the external atoms and a triplet resonance at −552.0 ppm corresponding to the central P atom. We notice that while the attachment of Fe(CO)_4_ or Me^+^ fragments at the external P atom of the triphoshorus ligand in **2** causes a shielding effect on the central P atom, attachment of a Fe(CO)_4_ fragment at the latter site has a strong deshielding effect on it (by some 170 ppm, if compared with **9-Fe**; see below). This effect largely exceeds the coordination shifts for conventional PR_3_ donors, which usually fall within the range of +20 to +70 ppm.^36^

The presence of two Fe(CO)_4_ fragments in compound **9-Fe** is first indicated by its IR spectrum, which displays two high-frequency bands at 2046 (sh, m) and 2038 (vs) cm^–1^ corresponding to symmetric stretches of these fragments,^32^ in addition to the asymmetric ones (see the Experimental Section). Moreover, because the ^31^P NMR spectrum of this complex displays one doublet resonance at 417.8 ppm for the external P atom of the P_3_ ligand, it is concluded that both iron fragments are bound to these atoms, as substantiated crystallographically for the related W(CO)_5_ derivative of **2** (see below). The binding of these iron fragments at the external P atoms of the P_3_ chain of **2** causes a large shielding effect of almost 100 ppm on the central P atom, which now gives rise to a triplet resonance at −721.8 ppm. To our knowledge, this is the lowest chemical shift reported to date for a phosphorus-containing compound of any kind.^37^ As observed for compounds **8** and **10**, neither the P–P couplings in the P_3_ ligand nor the chemical shift of the P^*t*^Bu_2_ ligand in **9-Fe** has been much affected by the attachment of the Fe fragments. All of this suggests that the binding of Fe(CO)_4_ fragments at either the terminal or central P atoms of the triphoshorus ligand of **2** has only a modest effect on the P–P and Mo–Mo bonding of the complex. We finally note that the ^13^C NMR spectrum of **9-Fe**, in addition to the expected resonances for the equivalent pairs of Cp and ^*t*^Bu groups, displays just a doublet resonance at 214.8 ppm (*J*_PC_ = 6 Hz) for the Fe-bound carbonyls. This indicates the operation of fast local exchange between the axial and equatorial carbonyls at the trigonal-pyramidal Fe(CO)_4_P fragments of the molecule, not investigated.

### Addition of M(CO)_5_ and M(CO)_4_ Fragments (M = Mo, W) to Complex **2**

**Scheme 4:**
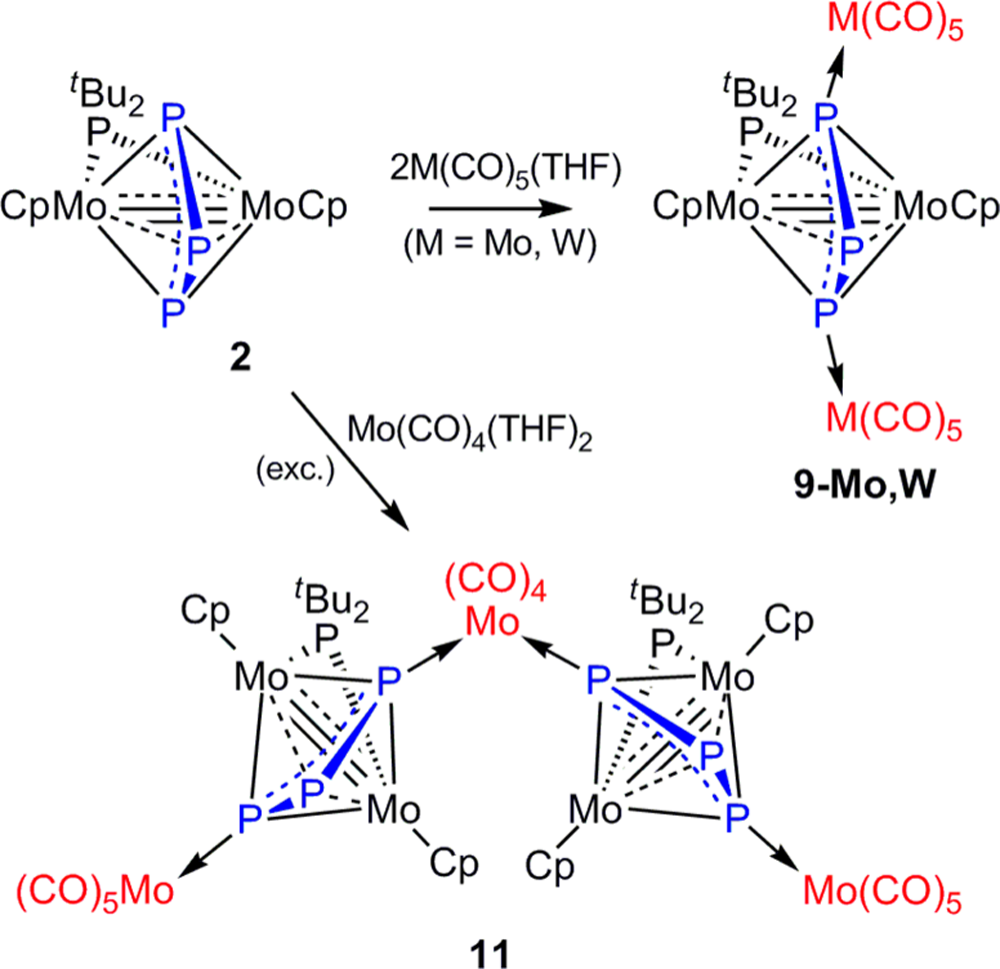
Molybdenum and Tungsten Derivatives of Complex **2**

To prepare molybdenum and tungsten analogues of compounds **8**, **9-Fe**, and **10**, we investigated the reactions of **2** with the tetrahydrofuran complexes [M(CO)_5_(THF)] (M = Mo, W). These reactions take place rapidly in toluene solution (see the Experimental section) to give the corresponding tetranuclear derivatives [Mo_2_M_2_Cp_2_(μ-η^3^:η^3^:κ^1^:κ^1^-P_3_)(μ-P^*t*^Bu_2_)(CO)_10_] (M = Mo(**9-Mo**), W(**9-W**)) in all cases (Scheme 4). Attempts to even detect analogues of compounds **8** and **10** (by using defect or excess [M(CO)_5_(THF)] in these reactions) were unsuccessful. We next investigated the reactions of **2** with the tetracarbonyl adducts [M(CO)_4_(THF)_2_] (M = Mo, W), since previous work from our lab have shown that 14-electron metal carbonyl fragments M(CO)_4_ can eventually insert into the P–P bond of the diphosphenyl complex [Mo_2_Cp_2_(μ-η^2^:η^2^-P_2_Me)(μ-PCy_2_)(CO)_2_].^11^ Unfortunately, reaction of **2** with complexes [M(CO)_4_(THF)_2_] gave in both cases the corresponding tetranuclear derivative of type **9** as the major product, and we could only isolate a new product incorporating a M(CO)_4_ fragment from the reaction with the Mo adduct, even if in modest yield (14%). The latter has been identified as the heptanuclear derivative [Mo_7_Cp_4_(μ-η^3^:η^3^:κ^1^:κ^1^-P_3_)_2_(μ-P^*t*^Bu_2_)_2_(CO)_14_] (**11**).

### Structure of Tetranuclear Derivatives **9-Mo** and **9-W**

**Figure 4:**
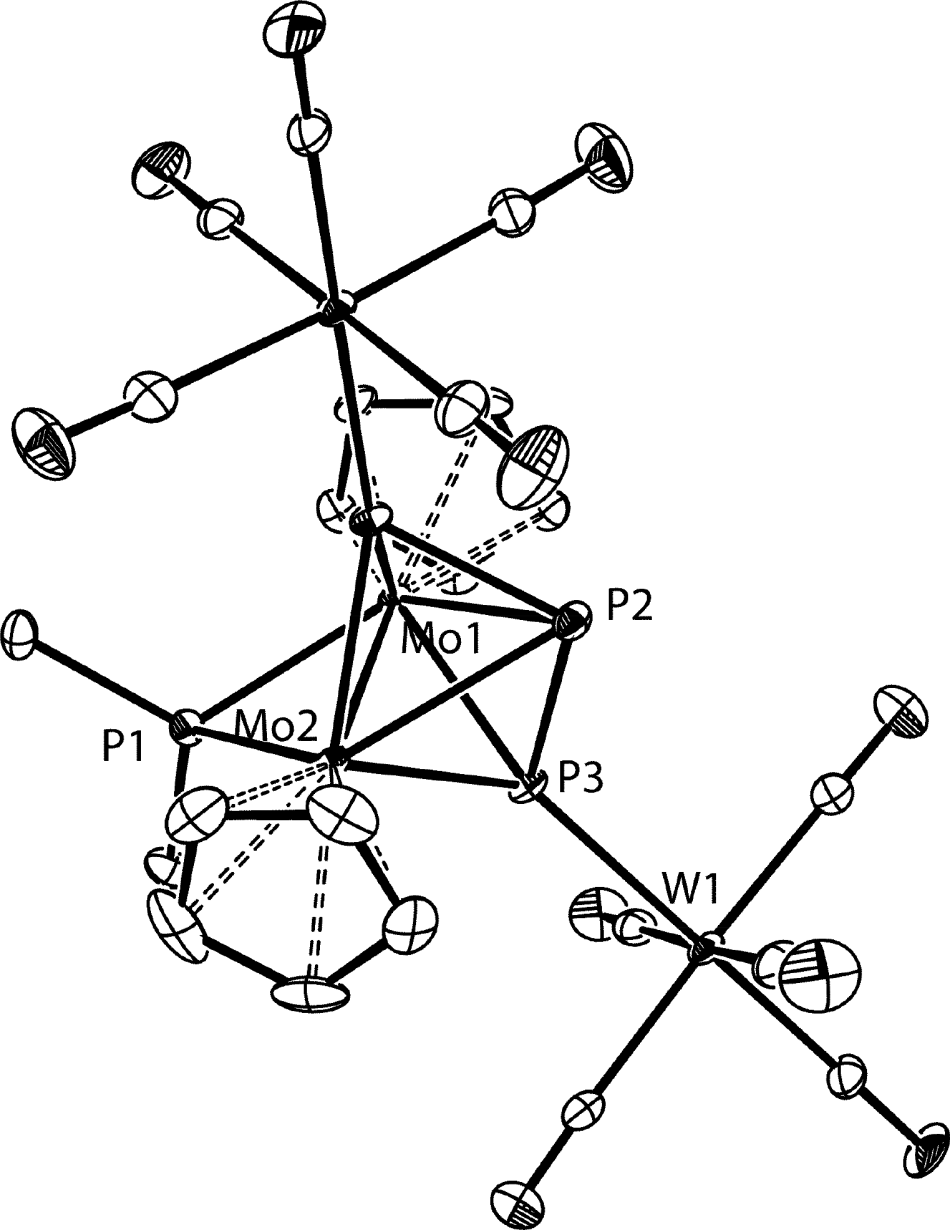
ORTEP diagram (30% probability) of compound **9-W**, with ^*t*^Bu groups (except their C^1^ atoms) and H atoms omitted for clarity.

The molecular structure of **9-W** in the crystal (Figure 4 and Table 3) can be derived from that of **2** upon attachment of a W(CO)_5_ fragment at each of the external P atoms of the P_3_ ligand. The resulting environment around the W atoms is octahedral as expected, and the W–P lengths of 2.4927(8) Å are a bit longer than that measured in the phosphide complex [Mo_2_WCp_2_(μ_3_-P)(μ-PCy_2_)(CO)_7_] (2.457(3) Å),^11a^ perhaps denoting some steric pressure from the bulky P^*t*^Bu_2_ ligand. The overall positioning of the W(CO)_5_ fragments in **9-W** is similar to the one computed for the methyl group in **7**, that is, with the W atom almost placed in the corresponding Mo_2_P plane, then completing a distorted trigonal-pyramidal environment around the external P atoms of the triphosphorus ligand. On the other hand, the geometrical parameters within the central Mo_2_P_3_ backbone in **9-W** are similar to those measured in **2**, with small elongations being observed for the intermetallic length (2.6558(5) Å) as well as the P–P and Mo–P(central) separations (ca. 2.17 and 2.68 Å, respectively), while the Mo–P(external) distances (ca. 2.37 Å) are slightly contracted. These geometrical modifications are qualitatively analogous to the ones following methylation of **2**, as computed for the cation in complex **7** (Figure 3).

**Table 3:** Selected Bond Lengths (Å) and Angles (deg) for Compound **9-W**

Mo1–Mo2	2.6558(5)	Mo1–P1–Mo2	66.07(3)
Mo1–P1	2.440(1)	Mo1–P2–Mo2	59.56(3)
Mo2–P1	2.432(1)	Mo1–P3–Mo2	68.05(2)
Mo1–P2	2.675(1)	W1–P3–P2	120.78(5)
Mo2–P2	2.672(2)	P1–Mo1–P2	116.99(4)
Mo1–P3	2.3754(8)	P1–Mo2–P2	117.38(4)
Mo2–P3	2.3708(9)	P1–Mo1–P3	92.19(3)
W1–P3	2.4927(8)	P1–Mo2–P3	92.51(3)
P2–P3	2.169(1)	P3–P2–P3′	103.65(7)

Spectroscopic data in solution for compounds **9-Mo** and **9-W** (Table 1 and Experimental Section) are consistent with the symmetrical structure found in the crystal for **9-W**. The presence of two M(CO)_5_ fragments attached to the triphosphorus ligand is first indicated by its IR spectrum, which displays two-high frequency bands at ca. 2071 (w, sh) and 2064 (m) cm^–1^ corresponding to symmetric C–O stretches of these fragments,^32^ in addition to the asymmetric ones (see the Experimental Section). Besides this, the ^31^P NMR spectra of these complexes display in each case just one doublet resonance for the external atoms of the P_3_ ligand, with a chemical shift expectedly sensitive to the metal in the carbonyl fragment (δ_P_ 391.6 and 354.3 ppm for the Mo and W derivatives, respectively). In contrast, the central atom of the P_3_ ligand gives rise to a triplet resonance at ca. −690 ppm in both complexes, a position ca. 65 ppm more shielded than in the parent compound **2**. The P–P coupling (ca. 390 Hz) is only marginally lower than in the parent complex (405 Hz), which is in agreement with the very modest lengthening of ca. 0.02 Å observed for the P–P bonds in **9-W**. Finally, we note that the resonance for the P^*t*^Bu_2_ ligand in these complexes appears at ca. 185 ppm, as also observed for **9-Fe** and for compounds **8** and **10**, and not far from the position observed for the parent **2**. This suggests that the intermetallic interaction is not severely modified by the coordination of the M(CO)_*n*_ fragments at the triphosphorus ligand in these species, in agreement with the very modest lengthening of just 0.03 Å observed for the intermetallic separation in **9-W**, when compared to the parent complex **2**.

### Structure of the Heptanuclear Derivative **11**

**Figure 5:**
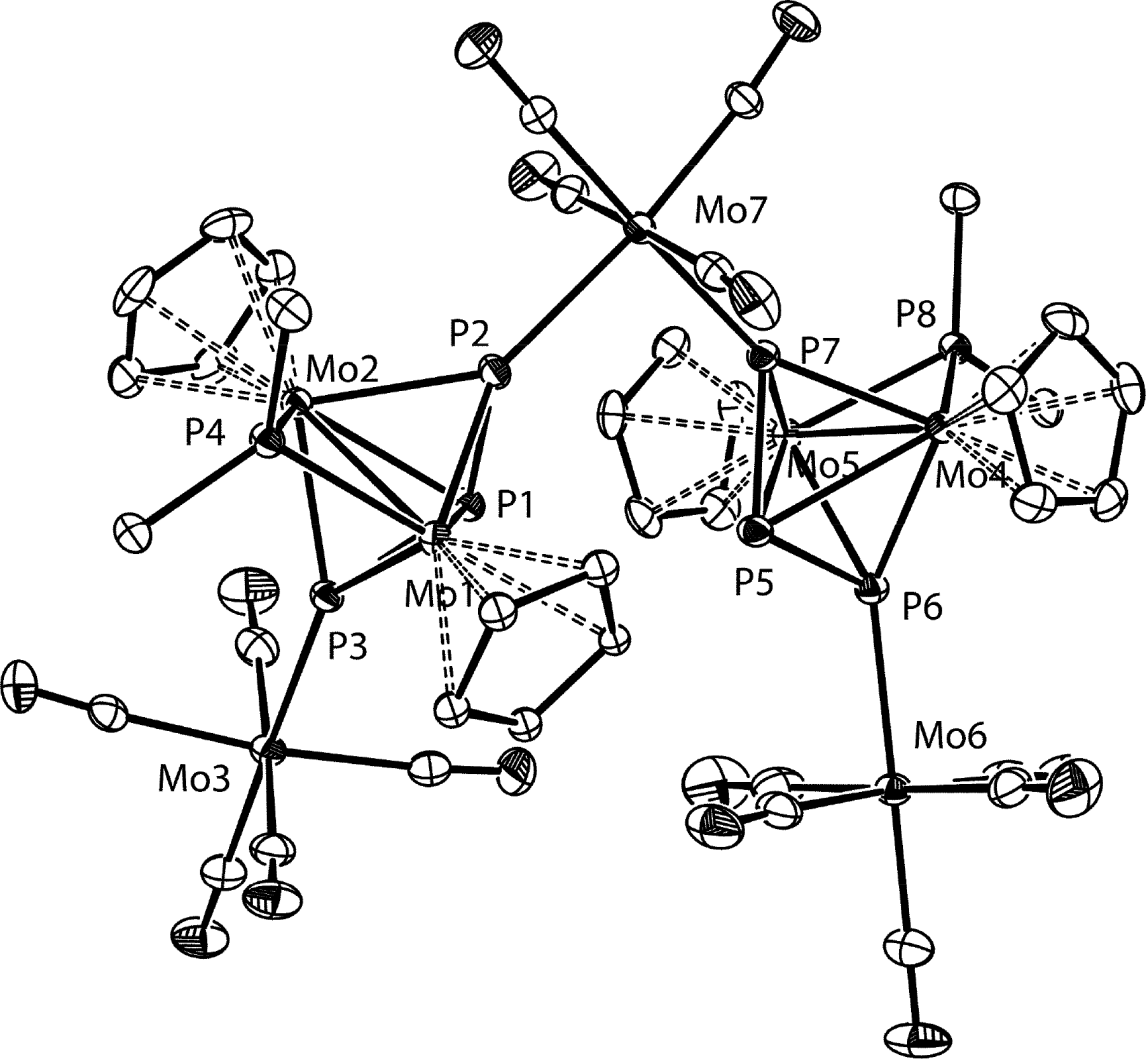
ORTEP diagram (30% probability) of compound **11**, with ^*t*^Bu groups (except their C^1^ atoms) and H atoms omitted for clarity.

The molecular structure of compound **11** in the crystal (Figure 5) can be viewed as built from two molecules of the parent compound **2** connected to a Mo(CO)_4_ fragment through their external P atoms, to render a *cis*-Mo(CO)_4_P_2_ octahedral environment around the bridging Mo atom (P–Mo–P = 95.78(4)°). In addition, each Mo_2_P_3_ subunit bears an additional Mo(CO)_5_ fragment at the remaining external P atom of the P_3_ ligand, thus rendering a local environment comparable to the one observed for the tungsten complex **9-W**. In fact, the geometrical parameters within these Mo_2_P_3_ subunits (Table 4) are very similar to those measured for **9-W** and therefore deserve no additional comments.

**Table 4:** Selected Bond Lengths (Å) and Angles (deg) for Compound **11**

Mo1–Mo2	2.6561(7)	Mo1–P1–Mo2	59.63(3)
Mo1–P1	2.670(1)	Mo1–P2–Mo2	67.63(3)
Mo1–P2	2.392(1)	Mo1–P3–Mo2	68.04(3)
Mo1–P3	2.375(1)	Mo3–P3–P1	116.09(6)
Mo1–P4	2.431(1)	Mo7–P2–P1	120.82(6)
Mo3–P3	2.484(1)	P4–Mo1–P1	117.14(4)
Mo7–P2	2.477(1)	P4–Mo1–P2	93.11(5)
P1–P2	2.149(2)	P4–Mo1–P3	92.16(4)
P1–P3	2.156(2)	P2–P1–P3	104.56(7)
Mo4–Mo5	2.6545(5)	Mo4–P5–Mo5	60.01(3)
Mo4–P5	2.664(1)	Mo4–P6–Mo5	67.79(3)
Mo4–P6	2.376(1)	Mo4–P7–Mo5	67.38(3)
Mo4–P7	2.384(1)	Mo6–P6–P5	114.82(6)
Mo4–P8	2.418(1)	Mo7–P7–P5	121.94(6)
Mo6–P6	2.529(1)	P8–Mo4–P5	116.93(4)
Mo7–P7	2.509(1)	P8–Mo4–P6	92.33(4)
P5–P6	2.164(2)	P8–Mo4–P7	93.70(4)
P5–P7	2.148(2)	P6–P5–P7	105.50(7)
		P2–Mo7–P7	95.78(4)

Spectroscopic data in solution for compound **11** (Table 1 and Experimental Section) are consistent with retention of the structure found in the crystal. First, its IR spectrum now displays, in addition to a high-frequency band at 2068 cm^–1^ to be expected for the symmetric C–O stretch of the Mo(CO)_5_ fragments, a medium-intensity band at 2015 cm^–1^ that we can assign to the symmetric C–O stretch of a cisoid or *C*_2*v*_ M(CO)_4_ fragment.^32^ Moreover, the ^31^P spectrum of **11** indicates that both Mo_2_P_3_ subunits are equivalent in solution, with chemical shifts and P–P couplings comparable to those measured for the tetranuclear molybdenum complex **9-Mo**. Of course, the external atoms of the P_3_ ligand are now inequivalent, but their chemical shifts (δ_P_ 383.8 and 403.8 ppm) are similar to each other, so assignment of these resonances to either Mo(CO)_5_- or Mo(CO)_4_-bound P atoms is not obvious. Other spectroscopic features for **11** are as expected and deserve no particular comment.

## Concluding Remarks

Reaction of the methyl-bridged complex [Mo_2_Cp_2_(μ-κ^1^:η^2^-CH_3_)(μ-P^*t*^Bu_2_)(μ-CO)] (**1**) with P_4_ at 333 K involves formal elimination of methylphosphinidene as the dominant process, to give the triphosphorus-bridged complex [Mo_2_Cp_2_(μ-η^3^:η^3^-P_3_)(μ-P^*t*^Bu_2_)] (**2**), but there is also a minor side process involving insertion of a P_2_ unit into the Mo–Me bond of **1** that yields the diphosphenyl-bridged complex [Mo_2_Cp_2_(μ-η^2^:η^2^-P_2_Me)(μ-P^*t*^Bu_2_)(CO)_2_]. The latter is more conveniently prepared by using the synthetic procedure previously developed by us for related PCy_2_-bridged complexes, which in this case involves the room temperature reaction between the unsaturated anion Na[Mo_2_Cp_2_(μ-P^*t*^Bu_2_)(μ-CO)_2_] with P_4_ to give a diphosphorus-bridged intermediate Na[Mo_2_Cp_2_(μ-η^2^:η^2^-P_2_)(μ-P^*t*^Bu_2_)(CO)_2_], which is then reacted with MeI. According to our DFT calculations, the triphosphorus ligand in **2** can be described as an allylic-like P_3_^–^ ligand acting as a six-electron donor via the external P atoms, while the binding of the central P atom to the metal atoms, much weaker, derives from ligand-to-metal and metal-to-ligand interactions involving the π and π* orbitals of the P_3_^–^ ligand, both of them having a weakening effect on both the Mo–Mo and P–P connections. As result of all of it, the intermetallic bond in **2** has geometric and topological properties intermediate between those of double and triple bonds, while the properties of the P–P bonds are intermediate between those of single and allylic-like interactions. In spite of the electronic unsaturation of the molecule, the chemical behavior of **2** is dominated by the electron-donor ability associated with the lone pairs located at the P atoms of the triphosphorus ligand, which can be easily methylated, and can also bind up to three metal–carbonyl fragments M(CO)_*n*_ (M = Fe, Mo, W), as shown by the formation of the triiron derivative [Mo_2_Fe_3_Cp_2_(μ-η^3^:η^3^:κ^1^:κ^1^:κ^1^-P_3_)(μ-P^*t*^Bu_2_)(CO)_12_]. In all these reactions, attachment of the corresponding electrophile at the external P atoms of the P_3_ chain is preferred. This causes little geometrical modifications on the Mo_2_P_3_ skeleton of the molecule but causes a considerable nuclear shielding of ca. 60–100 ppm on the central P atom of the ligand, maximum for the diiron derivative [Mo_2_Fe_2_Cp_2_(μ-η^3^:η^3^:κ^1^:κ^1^-P_3_)(μ-P^*t*^Bu_2_)(CO)_8_], which displays the corresponding resonance at −721.8 ppm, the lowest ^31^P chemical shift reported to date for a P-containing species.

## Experimental Section

### General Procedures and Starting Materials

All manipulations and reactions were performed under an argon (99.995%) atmosphere by using standard Schlenk techniques. Solvents were purified according to the literature procedures and distilled prior to use.^38^ Compound [Mo_2_Cp_2_(μ-κ^1^:η^2^-CH_3_)(μ-P^*t*^Bu_2_)(μ-CO)] (**1**) was prepared in situ through a slight modification of the method described previously,^13^ now involving irradiation with UV–vis light of toluene solutions of [Mo_2_Cp_2_(μ-κ^1^:η^2^-CH_3_)(μ-P^*t*^Bu_2_)(CO)_2_] at 288 K in a quartz jacketed Schlenk tube (ca. 40 min for 0.100 g of dicarbonyl complex) and used without further purification by assuming a 100% yield. Tetrahydrofuran solutions of Na[Mo_2_Cp_2_(μ-P^*t*^Bu_2_)(μ-CO)_2_] (**4**)^13^ and [M(CO)_5_(THF)] (M = Mo, W)^39^ also were prepared in situ as reported previously. Modified literature procedures were employed in the preparation of the adducts [M(CO)_4_(THF)_2_],^40^ which were obtained at 288 K and short reaction times (Mo) or at 273 K and longer reaction times (W); IR monitoring was used to determine the optimum reaction time in each case. All other reagents were obtained from commercial suppliers and used as received, unless otherwise stated. Petroleum ether refers to that fraction distilling in the range 338–343 K. Filtrations were performed through diatomaceous earth unless otherwise stated. Chromatographic separations were performed by using jacketed columns cooled by tap water (ca. 288 K) or by a closed 2-propanol circuit kept at the desired temperature with a cryostat. Commercial aluminum oxide (activity I, 70–290 mesh) was degassed under vacuum prior to use. The latter was mixed under argon with the appropriate amount of water to reach activity IV. IR stretching frequencies of CO ligands are measured in solution (using CaF_2_ windows), are termed ν(CO), and are given in wavenumbers (cm^–1^). Nuclear magnetic resonance (NMR) spectra were routinely recorded at 295 K unless otherwise stated. Chemical shifts (δ) are given in ppm, relative to internal tetramethylsilane (^1^H, ^13^C), or external 85% aqueous H_3_PO_4_ solutions (^31^P). Coupling constants (*J*) are given in hertz. Labels for P atoms in P_3_ units are given according to the figure in Table 1.

### Preparation of [Mo_2_Cp_2_(μ-η^3^:η^3^-P_3_)(μ-P^*t*^Bu_2_)] (**2**)

A toluene solution of P_4_ (1.5 mL of a 0.18 M solution, 0.27 mmol) was added to a toluene solution (5 mL) containing ca. 0.186 mmol of compound **1**, prepared in situ from [Mo_2_Cp_2_(μ-κ^1^:η^2^-CH_3_)(μ-P^*t*^Bu_2_)(CO)_2_] (0.100 g, 0.186 mmol), and the mixture was stirred at 333 K for 15 min to give a black solution. The solvent was then removed under vacuum, the residue was extracted with dichloromethane/petroleum ether (1/6), and the extracts were filtered by using a cannula; then the filtrate was chromatographed on alumina at 243 K. Elution with the same solvent mixture gave a blue fraction yielding, after removal of solvents, compound **2** as a blue solid (0.086 g, 83%). Elution with dichloromethane/petroleum ether (1/3) gave a minor orange fraction yielding analogously small amounts of compound [Mo_2_Cp_2_(μ-η^2^:η^2^-P_2_Me)(μ-P^*t*^Bu_2_)(CO)_2_] (**3**) (0.006 g, 4%). Data for **2**: Anal. Calcd for C_18_H_28_Mo_2_P_4_: C, 38.59; H, 5.04. Found: C, 38.32; H, 5.13. ^31^P{^1^H} NMR (162.19 MHz, C_6_D_6_): δ 412.0 [d, *J*_PP_ = 405, P^1^(P_3_)], 176.1 (s, μ-P^*t*^Bu_2_), −626.5 [t, *J*_PP_ = 405, P^2^(P_3_)]. ^1^H NMR (400.54 MHz, C_6_D_6_): δ 4.83 (s, 10H, Cp), 1.03 (d, *J*_HP_ = 14, 18H, ^*t*^Bu). ^13^C{^1^H} NMR (100.72 MHz, C_6_D_6_): δ 88.4 (s, Cp), 40.5 [d, *J*_CP_ = 13, C^1^(^*t*^Bu)], 35.7 [s, C^2^(^*t*^Bu)].

### Preparation of Tetrahydrofuran Solutions of Na[Mo_2_Cp_2_(μ-η^2^:η^2^-P_2_)(μ-P^*t*^Bu_2_)(CO)_2_] (**5**)

A toluene solution of P_4_ (0.75 mL of a 0.18 M solution, 0.135 mmol) was added to a Schlenk tube equipped with a Young’s valve. The solvent was removed under vacuum, and then 15 mL of a tetrahydrofuran suspension of freshly prepared compound **4** (ca. 0.1 mmol) was added by using a cannula; the mixture was stirred at room temperature for 4 h to give an orange-brown solution containing compound **5** as unique organometallic species. This air-sensitive solution was used without further purification. ν(CO) (THF): 1833 (vs), 1758 (w), 1695 (s). ^31^P{^1^H} NMR (THF): 222.9 (s, μ-P^*t*^Bu_2_), −156.9 (s, μ-P_2_).

### Preparation of [Mo_2_Cp_2_(μ-η^2^:η^2^-P_2_Me)(μ-P^*t*^Bu_2_)(CO)_2_] (**3**)

Neat MeI (50 μL, 0.803 mmol) was added to the tetrahydrofuran solution of compound **5** (ca. 0.1 mmol) prepared as described above and cooled at 273 K, and the mixture was stirred at this temperature for 40 min to give a brown-yellowish solution. The solvent was then removed under vacuum, the residue was extracted with dichloromethane/petroleum ether (1/4), and the extracts were chromatographed on alumina at 253 K. Elution with the same solvent mixture gave first a minor yellow fraction yielding, after removal of solvents, complex [Mo_2_Cp_2_(μ-P^*t*^Bu_2_)(μ-PH_2_)(CO)_2_] (**6**) as a yellow solid (0.008 g, 14%) and then a major orange fraction yielding analogously compound **3** as a red-orange solid (0.035 g, 58%). Data for **3**: Anal. Calcd for C_21_H_31_Mo_2_O_2_P_3_: C, 42.02; H, 5.21. Found: C, 41.83; H, 5.39. ν(CO) (CH_2_Cl_2_): 1884 (s), 1801 (vs). ^31^P{^1^H} NMR (121.48 MHz, C_6_D_6_): δ 223.5 (dd, *J*_PP_ = 19, 8, μ-P^*t*^Bu_2_), −84.9 (dd, *J*_PP_ = 524, 19, μ-PMe), −286.0 (dd, *J*_PP_ = 524, 8, μ-P). ^31^P NMR (121.48 MHz, C_6_D_6_): δ 223.5 (m, μ-P^*t*^Bu_2_), −84.9 (dm, *J*_PP_ = 524, μ-PMe), −286.0 (dd, *J*_PP_ = 524, 8, μ-P). ^1^H NMR (300.13 MHz, C_6_D_6_): δ 5.12 (d, *J*_HP_ = 2, 5H, Cp), 4.86 (s, 5H, Cp), 1.56 (dd, *J*_HP_ = 12, 3, 3H, PMe), 1.25, 1.16 (2d, *J*_HP_ = 12, 2 × 9H, ^*t*^Bu). ^13^C{^1^H} NMR (121.48 MHz, CD_2_Cl_2_): δ 242.4 (dt, *J*_CP_ = 40, 6, MoCO), 238.8 (ddd, *J*_CP_ = 28, 8, 4, MoCO), 88.0 (s, Cp), 85.9 (d, *J*_CP_ = 2, Cp), 44.0 [dd, *J*_CP_ = 8, 2, C^1^(^*t*^Bu)], 42.3 [d, *J*_CP_ = 4, C^1^(^*t*^Bu)], 34.1 [d, *J*_CP_ = 3, C^2^(^*t*^Bu)], 33.7 [dd, *J*_CP_ = 8, 4, C^2^(^*t*^Bu)], −6.6 (dt, *J*_CP_ = 22, 4, PMe). Data for **6**: ν(CO) (CH_2_Cl_2_): 1875 (w, sh), 1835 (vs). ^31^P{^1^H} NMR (121.48 MHz, C_6_D_6_): δ 175.8 (d, *J*_PP_ = 7, μ-P^*t*^Bu_2_), −55.5 (d, *J*_PP_ = 7, μ-PH_2_). ^31^P NMR (121.48 MHz, C_6_D_6_): δ 175.8 (m, μ-P^*t*^Bu_2_), −55.5 (t, *J*_PP_ = 363, μ-PH_2_). ^1^H NMR (300.13 MHz, C_6_D_6_): δ 5.09 (s, 10H, Cp), 4.71 (dd, *J*_HP_ = 363, 3, 2H, PH_2_), 1.26 (d, *J*_HP_ = 13, 18H, ^*t*^Bu).

### Preparation of [Mo_2_Cp_2_(μ-η^3^:η^3^-P_3_Me)(μ-P^*t*^Bu_2_)](CF_3_SO_3_) (**7**)

Neat CF_3_SO_3_Me (10 μL, 0.088 mmol) was added to a dichloromethane solution (8 mL) of compound **2** (0.020 g, 0.036 mmol) at 253 K, and the mixture was stirred at that temperature for 15 min and then allowed to reach room temperature for 10 min to give a green solution. The solvent was then removed under vacuum, and the residue was washed with petroleum ether (5 × 3 mL) and dried under vacuum to yield compound **7** as a quite pure green solid (0.022 g, 84%; see the Supporting Information for spectra). All attempts to further purify this solid through crystallization techniques, however, resulted in its progressive decomposition, so no microanalytical data were obtained for this product. ^31^P{^1^H} NMR (121.48 MHz, CD_2_Cl_2_): δ 444.3 [dd, *J*_PP_ = 423, 21, P^3^(P_3_)], 315.4 [dt, *J*_PP_ = 410, 21, MeP^1^(P_3_)], 188.7 (dd, *J*_PP_ = 21, 8, μ-P^*t*^Bu_2_), −699.5 [ddd, *J*_PP_ = 423, 410, 8, P^2^(P_3_)]. ^31^P NMR (121.48 MHz, CD_2_Cl_2_): δ 444.3 [dd, *J*_PP_ = 423, 21, P^3^(P_3_)], 315.4 [dm, *J*_PP_ = 410, MeP^1^(P_3_)], 188.7 (m, μ-P^*t*^Bu_2_), −699.5 [t, br, *J*_PP_ = 416, P^2^(P_3_)]. ^1^H NMR (300.13 MHz, CD_2_Cl_2_): δ 5.79 (s, 10H, Cp), 3.36 (dd, *J*_HP_ = 12, 7, 3H, PMe), 1.28, 0.91 (2d, *J*_HP_ = 15, 2 × 9H, ^*t*^Bu). ^31^C{^1^H} NMR (100.62 MHz, CD_2_Cl_2_): δ 93.9 (s, Cp), 43.0 [d, *J*_CP_ = 14, C^1^(^*t*^Bu)], 39.4 [d, *J*_CP_ = 11, C^1^(^*t*^Bu)], 35.9 [t, *J*_CP_ = 4, C^2^(^*t*^Bu)], 35.3 [dd, *J*_CP_ = 7, 4, C^2^(^*t*^Bu)], 12.6 [dd, *J*_CP_ = 10, 8, PMe].

### Preparation of Solutions of [Mo_2_FeCp_2_(μ-η^3^:η^3^:κ^1^-P_3_)(μ-P^*t*^Bu_2_)(CO)_4_] (**8**)

Solid [Fe_2_(CO)_9_] (0.015 g, 0.041 mmol) was added to a toluene solution (5 mL) of compound **2** (0.030 g, 0.054 mmol), and the mixture was stirred for 10 min, whereby all iron reagent was consumed. The ^31^P{^1^H} NMR spectrum of this solution denoted the presence of unreacted **2**, along with compounds **8** and **9-Fe** in a ca. 3:4:1 ratio (see the Supporting Information). Attempts to isolate compound **8** from this mixture upon chromatography yielded only complex **9-Fe** (see below). ^31^P{^1^H} NMR (121.48 MHz, toluene): δ 455.2 [dt, *J*_PP_ = 405, 13, P^3^(P_3_)], 377.6 [dd, *J*_PP_ = 420, 10, P^1^(P_3_)], 179.8 (m, μ-P^*t*^Bu_2_), −648.5 [ddd, *J*_PP_ = 420, 405, 8, P^2^(P_3_)].

### Preparation of [Mo_2_Fe_2_Cp_2_(μ-η^3^:η^3^:κ^1^:κ^1^-P_3_)(μ-P^*t*^Bu_2_)(CO)_8_] (**9-Fe**)

Solid [Fe_2_(CO)_9_] (0.041 g, 0.113 mmol) was added to a toluene solution (5 mL) of compound **2** (0.030 g, 0.054 mmol), and the mixture was stirred for 10 min to give a green solution containing compound **9-Fe** as major product, along with small amounts of the triiron complex [Mo_2_Fe_3_Cp_2_(μ-η^3^:η^3^:κ^1^:κ^1^:κ^1^-P_3_)(μ-P^*t*^Bu_2_)(CO)_12_] (**10**). The latter, however, decomposed progressively to yield **9-Fe** and could not be isolated. The solvent was then removed under vacuum, the residue was extracted with dichloromethane/petroleum ether (1/6), and the extracts were chromatographed on alumina at 253 K. Elution with the same solvent mixture gave a green fraction yielding, after removal of solvents, complex **9-Fe** as a green solid (0.045 g, 93%). Data for compound **9-Fe**: Anal. Calcd for C_26_H_28_Fe_2_Mo_2_O_8_P_4_: C, 34.85; H, 3.15. Found: C, 35.18; H, 3.08. ν(CO) (CH_2_Cl_2_): 2046 (sh, m), 2038 (vs), 1973 (m), 1946 (s). ^31^P{^1^H} NMR (121.48 MHz, C_6_D_6_): δ 417.8 [dd, *J*_PP_ = 422, 16, P^1^(P_3_)], 185.7 (dd, *J*_PP_ = 16, 11, μ-P^*t*^Bu_2_), −721.8 [td, *J*_PP_ = 422, 11, P^2^(P_3_)]. ^1^H NMR (400.13 MHz, C_6_D_6_): δ 5.21 (s, 10H, Cp), 0.77 (d, *J*_HP_ = 15, 18H, ^*t*^Bu). ^13^C{^1^H} NMR (100.63 MHz, C_6_D_6_): δ 214.8 (d, *J*_CP_ = 6, FeCO), 92.8 (s, Cp), 40.6 [d, *J*_CP_ = 14, C^1^(^*t*^Bu)], 35.0 [d, *J*_CP_ = 4, C^2^(^*t*^Bu)]. Data for compound **10**: ^31^P{^1^H} NMR (121.48 MHz, toluene): δ 404.3 [dd, *J*_PP_ = 445, 16, P^1^(P_3_)], 177.0 (q, *J*_PP_ = 16, μ-P^*t*^Bu_2_), −552.0 [td, *J*_PP_ = 445, 16, P^2^(P_3_)].

### Preparation of [Mo_4_Cp_2_(μ-η^3^:η^3^:κ^1^:κ^1^-P_3_)(μ-P^*t*^Bu_2_)(CO)_10_] (**9-Mo**)

A tetrahydrofuran solution (5 mL) of [Mo(CO)_5_(THF)] was prepared in situ from [Mo(CO)_6_] (0.030 g, 0.114 mmol) and then added to compound **2** (0.030 g, 0.054 mmol). The solvent was then removed under vacuum, the residue was dissolved in toluene (5 mL), and the mixture was stirred at room temperature for 5 min to give a green solution. The solvent was again removed under vacuum, the residue was extracted with dichloromethane/petroleum ether (1/8), and the extracts were chromatographed on alumina at 288 K. Elution with dichloromethane/petroleum ether (1/4) gave a green fraction yielding, after removal of solvents, complex **9-Mo** as a green solid (0.051 g, 91%). Anal. Calcd for C_28_H_28_Mo_4_O_10_P_4_: C, 32.58; H, 2.73. Found: C, 32.96; H, 3.05. ν(CO) (CH_2_Cl_2_): 2071 (w, sh), 2064 (m), 1950 (vs), 1931 (m, sh). ^31^P{^1^H} NMR (121.48 MHz, CD_2_Cl_2_): δ 391.6 [dd, *J*_PP_ = 394, 14, P^1^(P_3_)], 184.2 (q, *J*_PP_ = 12, μ-P^*t*^Bu_2_), −687.3 [td, *J*_PP_ = 394, 10, P^2^(P_3_)]. ^1^H NMR (300.13 MHz, CD_2_Cl_2_): δ 5.49 (s, 10H, Cp), 1.17 (d, *J*_HP_ = 15, 18H, ^*t*^Bu).

### Preparation of [Mo_2_W_2_Cp_2_(μ-η^3^:η^3^:κ^1^:κ^1^-P_3_)(μ-P^*t*^Bu_2_)(CO)_10_] (**9-W**)

The procedure is analogous to the one described above for **9-Mo**, but now with a tetrahydrofuran solution (5 mL) of [W(CO)_5_(THF)] prepared in situ from [W(CO)_6_] (0.040 g, 0.114 mmol). The chromatography was now performed at 253 K. Elution with dichloromethane/petroleum ether (1/8) gave a green fraction yielding, after removal of solvents, complex **9-W** as a green solid (0.057 g, 87%). The crystals used in the X-ray diffraction study were grown by the slow diffusion of a layer of petroleum ether into a concentrated tetrahydrofuran solution of the complex at 253 K. Anal. Calcd for C_28_H_28_Mo_2_O_10_P_4_W_2_: C, 27.84; H, 2.34. Found: C, 27.59; H, 2.62. ν(CO) (CH_2_Cl_2_): 2070 (w, sh), 2063 (m), 1943 (vs), 1923 (m, sh). ^31^P{^1^H} NMR (121.48 MHz, CD_2_Cl_2_): δ 354.3 [dd, *J*_PP_ = 391, 15, P^1^(P_3_)], 185.2 (td, *J*_PP_ = 15, 11, μ-P^*t*^Bu_2_), −689.8 [td, *J*_PP_ = 391, 11, P^2^(P_3_)]. ^1^H NMR (300.13 MHz, CD_2_Cl_2_): δ 5.55 (s, 10H, Cp), 1.17 (d, *J*_HP_ = 15, 18H, ^*t*^Bu). ^13^C{^1^H} NMR (100.63 MHz, CD_2_Cl_2_): δ 201.1 (AXX′ mult, *J*_CP_ + *J*_CP′_ = 26, WCO_ax_), 197.6 [q, *J*_CP_ = 4, *J*_CW_ = 126, WCO_eq_], 91.8 (s, Cp), 41.3 [d, *J*_CP_ = 14, C^1^(^*t*^Bu)], 35.6 [s, C^2^(^*t*^Bu)].

### Preparation of [Mo_7_Cp_4_(μ-η^3^:η^3^:κ^1^:κ^1^-P_3_)_2_(μ-P^*t*^Bu_2_)_2_(CO)_14_] (**11**)

A tetrahydrofuran solution (10 mL) of [Mo(CO)_4_(THF)_2_] was prepared in situ from [Mo(CO)_6_] (0.044 g, 0.168 mmol) and then added to compound **2** (0.030 g, 0.054 mmol), and the mixture was stirred at room temperature for 1 h to give a green solution. After removal of the solvent under vacuum, the residue was extracted with dichloromethane/petroleum ether (1/8), and the extracts were chromatographed on alumina at 288 K. Elution with dichloromethane/petroleum ether (1/4) gave a major green fraction yielding, after removal of solvents, complex **9-Mo** as a green solid (0.039 g, 67%). Elution with dichloromethane/petroleum ether (1/2) gave a second green fraction yielding analogously complex **11** as a green solid (0.008 g, 16%). The crystals used in the X-ray diffraction study of **11** were grown by the slow diffusion of layers of tetrahydrofuran and petroleum ether into a concentrated dichloromethane solution of the complex at 253 K. Anal. Calcd for C_50_H_56_Mo_7_O_14_P_8_: C, 33.36; H, 3.14. Found: C, 32.96; H, 3.05. ν(CO) (CH_2_Cl_2_): 2068 (m), 2015 (m), 1943 (vs), 1925 (m, sh). ^31^P{^1^H} NMR (121.48 MHz, CD_2_Cl_2_): δ403.8 (d, br, *J*_PP_ = 414, P^3^(P_3_)], 383.8 [d, br, *J*_PP_ = 380, P^1^(P_3_)], 183.7 (m, μ-P^*t*^Bu_2_), −678.6 [t, br, *J*_PP_ = 390, P^2^(P_3_)]. ^1^H NMR (300.13 MHz, CD_2_Cl_2_): δ5.63 (s, 10H, Cp), 1.27, 1.17 (2d, *J*_HP_ = 15, 2 × 9H, ^*t*^Bu).

### X-ray Structure Determination of Compounds **9-W** and **11**

Data collection for these compounds was performed at ca. 155 K on an Oxford Diffraction Xcalibur Nova single crystal diffractometer using Cu Kα radiation. Images were collected at a 62 mm fixed crystal-detector distance by using the oscillation method, with 1.3° oscillation and variable exposure time per image. Data collection strategy was calculated with the program CrysAlis Pro CCD,^41^ and data reduction and cell refinement were performed with the program CrysAlis Pro RED.^41^ In both cases, an empirical absorption correction was applied by using the SCALE3 ABSPACK algorithm as implemented in the program CrysAlis Pro RED. Using the program suite WinGX,^42^ we solved the structures by Patterson interpretation and phase expansion using SHELXL2016^43^ and refined with full-matrix least-squares on *F*^2^ using SHELXL2016. In general, all non-hydrogen atoms were refined anisotropically, except for atoms involved in disorder, and all hydrogen atoms were geometrically placed and refined by using a riding model. In compound **9-W**, one ^*t*^Bu group was disordered and satisfactorily modeled over two positions with 0.55/0.45 occupancies. The disordered carbon atoms were refined isotropically, and this caused a B-level alert in the corresponding checkcif file. In compound **11**, one of the cyclopentadienyl rings was disordered, satisfactorily modeled over two positions with 0.5 occupancies. Other data for the refinements of these structures can be found in Table S1.

### Computational Details

All DFT calculations were performed by using the GAUSSIAN09 package^44^ and the M06L functional.^45^ A pruned numerical integration grid (99,590) was used for all the calculations via the keyword Int = Ultrafine. Effective core potentials and their associated double-ζ LANL2DZ basis set were used for Mo atoms.^46^ The light elements (P, C, and H) were described with the 6-31G* basis.^47^ Geometry optimizations were performed under no symmetry restrictions by using initial coordinates derived from the X-ray data. Frequency analysis was performed for all the stationary points to ensure that a minimum structure with no imaginary frequencies was achieved in each case. Molecular orbitals and vibrational modes were visualized by using the MOLEKEL program.^48^ The topological analysis of ρ was performed by using the MultiWFN program.^49^

## Supporting Information Available

Crystal data for compounds **9-W** and **11** (CCDC 2083094 and 2083095), results of DFT calculations, and IR and NMR spectra for all new compounds (PDF)
Cartesian coordinates for all computed species (XYZ)

## Supplementary Material

ic1c01552_si_001.pdf

ic1c01552_si_003.xyz

## Accession Codes

CCDC 2083094–2083095 contain the supplementary crystallographic data for this paper. These data can be obtained free of charge via www.ccdc.cam.ac.uk/data_request/cif, or by emailing data_request@ccdc.cam.ac.uk, or by contacting The Cambridge Crystallographic Data Centre, 12 Union Road, Cambridge CB2 1EZ, UK; fax: +44 1223 336033.
