# Supplementary material for: Electronic Structure and Donor Ability of an Unsaturated Triphosphorus-Bridged Dimolybdenum Complex
Source: Inorg Chem. 2021 Jul 19;60(15):11548–61. doi: 10.1021/acs.inorgchem.1c01552 (PMC8901102; doi:10.1021/acs.inorgchem.1c01552)
Supplement: Supplementary file 1 — ic1c01552_si_001.pdf [file ic1c01552_si_001.pdf]

Supporting Information

Electronic Structure and Donor Ability of an Unsaturated Triphosphorus-Bridged Dimolybdenum Complex.

M. Angeles Alvarez, Melodie Casado-Ruano, M. Esther García, Daniel García-Vivó,* Ana M. Guerra, and Miguel A. Ruiz.*

Departamento de Química Orgánica e Inorgánica/IUQOEM, Universidad de Oviedo, E-33071 Oviedo, Spain.

Table S1. Crystal Data for New Compounds

	**9-W**	**11**
mol formula	$C_{28}H_{28}Mo_2O_{10}P_4W_2$	$C_{50}H_{56}Mo_7O_{14}P_8$
mol wt	1207.94	1800.29
cryst syst	orthorhombic	monoclinic
space group	*P*nma	$P2_1$/c
radiation (λ, Å)	1.54184	1.54184
a, Å	17.6550(3)	9.8514(3)
b, Å	21.0145(3)	31.0917(8)
c, Å	9.77630(10)	21.3645(6)
α, deg	90	90
β, deg	90	103.227(3)
γ, deg	90	90
V, Å^3	3627.11(9)	6370.3(3)
Z	4	4
calcd density, g cm^{-3}	2.212	1.877
absorp coeff, mm^{-1}	19.067	13.314
temperature, K	145.0(1)	150.3(2)
θ range (deg)	4.21-69.73	3.55-69.96
index ranges (*h*, *k*, *l*)	–21, 15; –25, 24 –8, 11	–11, 11; –37, 37 –25, 26
no. of reflns collected	17216	64795
no. of indep reflns (R_{int})	3493(0.0337)	11925(0.0545)
reflns with $I > 2\sigma(I)$	3205	10861
R indexes [data with $I > 2\sigma(I)$][a]	R_1 = 0.0223 wR_2 = 0.0495[b]	R_1 = 0.0578 wR_2 = 0.1572[c]
R indexes (all data)[a]	R_1 = 0.0257 wR_2 = 0.0510[b]	R_1 = 0.0679 wR_2 = 0.1686[c]
GOF	1.130	1.109
no. of restraints/params	0 / 220	0 / 701
$\Delta\rho$(max., min.), eÅ^{-3}	–1.106 /0.585	–1.418 / 0.994
CCDC deposition no	2083094	2083095

[a] $R = \Sigma||F_o| - |F_c|| / \Sigma|F_o|$. $wR = [\Sigma w(|F_o|^2 - |F_c|^2)^2 / \Sigma w|F_o|^2]^{1/2}$. $w = 1/[\sigma^2(F_o^2) + (aP)^2 + bP]$ where $P = (F_o^2 + 2F_c^2)/3$. [b] a = 0.0243, b = 0.0000. [c] a = 0.0790, b = 11.4669.

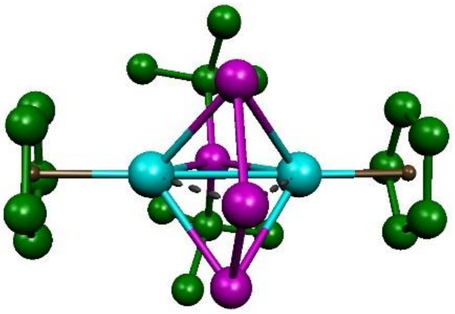

Figure S1. M06L-DFT optimized structure of complex **2**, with H atoms omitted for clarity. Selected bond lengths (Å): Mo–Mo = 2.633; Mo–P^{ext} = 2.411, 2.416, 2.411, 2.414; Mo–P^{int} = 2.662, 2.666; Mo–P^tBu_2 = 2.423, 2.424; P^{int}–P^{ext} = 2.164, 2.170. P–P–P = 107.35.

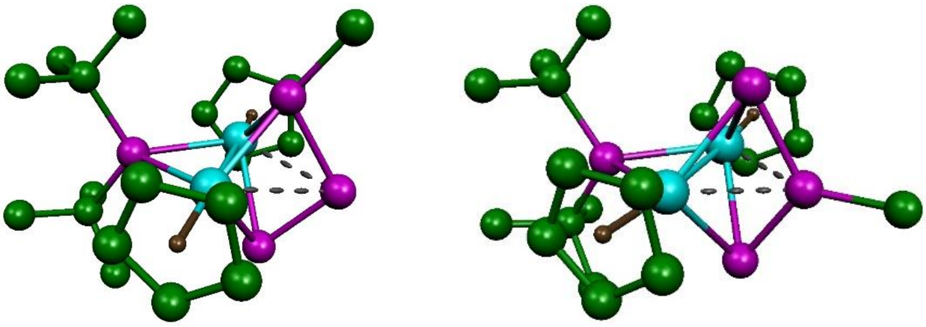

Figure S2. M06L-DFT optimized structure of the cation in complex **7** (left), and an isomer bearing the Me group at the internal P atom (P^{int}) of the P_3 chain (**7’**, right), with H atoms omitted for clarity. Selected averaged bond lengths for **7** (Å): Mo–Mo = 2.711; Mo–PMe = 2.343; Mo–P^{int} = 2.698; Mo–P = 2.431; Mo–P^tBu_2 = 2.447; PMe–P^{int}= 2.182; P^{int}–P= 2.152. Selected bond lengths for **7’**: Mo–Mo = 2.694; Mo–P^{ext} = 2.451; Mo–PMe = 2.547; Mo–P^tBu_2 = 2.442; P–PMe= 2.132. Relative Gibbs free energies at 298 K were 0 (**7**) and +54 kJ/mol (**7’**).

Figure S3. Selected M06L-DFT computed molecular orbitals of compound **2** viewed from a point close to the Mo–P(^{t}Bu$_2$)–Mo plane (left), with their energies (in eV) and main bonding character indicated below (LP stands for lone pair character at the P atoms). On the right, a view of these orbitals from a plane perpendicular to the above one.

	View 1	View 2
LUMO 113<br>$(\pi/\delta)^*_{MM}$<br>-2.79	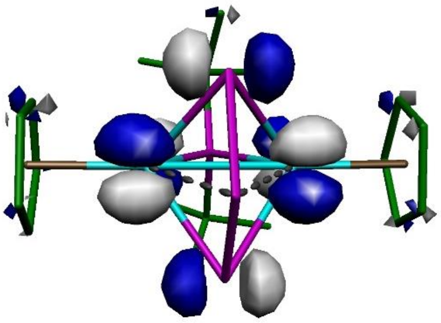	
Contributions (%)	Mo´s 68, P^{t}Bu$_2$ 1, P$_{ext}$ 21, P$_{cent}$ 0, Other 10	
HOMO 112<br>$(\pi/\delta)_{MM}$<br>-4.33	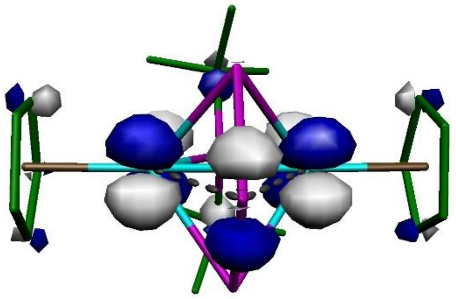	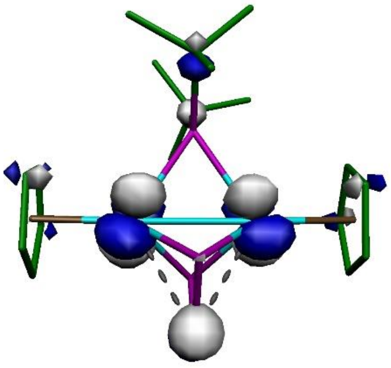
Contributions (%)	Mo´s 67, P^{t}Bu$_2$ 4, P$_{ext}$ 3, P$_{cent}$ 8, Other 19	
MO 111<br>$\sigma_{M2Pcent} + \delta^*_{MM}$<br>-4.70	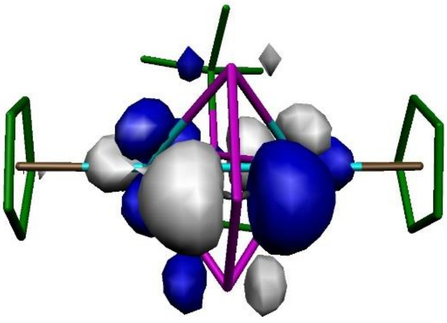	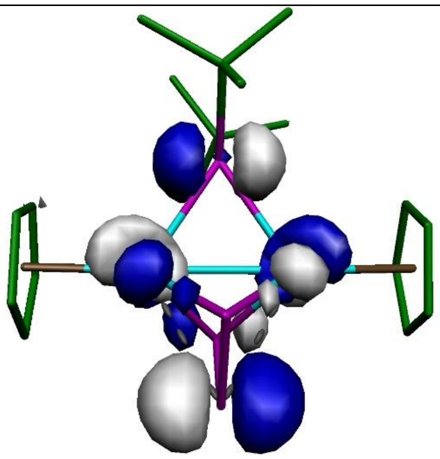
Contributions (%)	Mo´s 46, P^{t}Bu$_2$ 16, P$_{ext}$ 10, P$_{cent}$ 23, Other 5	

MO 110<br><br>σ_{MM} + LP<br><br>-4.86	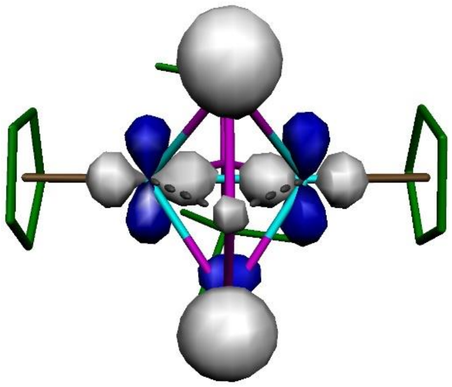	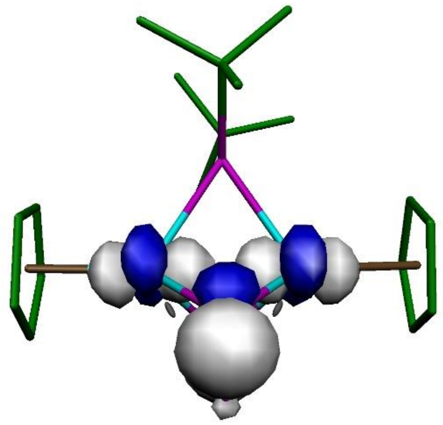
Contributions (%)	Mo´s 63, $P^{t}Bu_2$ 1, P_{ext} 30, P_{cent} 1, Other 5	
MO 109<br><br>σ_{M2Pext} + LP<br><br>-5.53	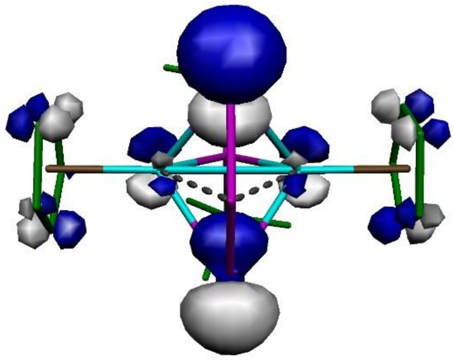	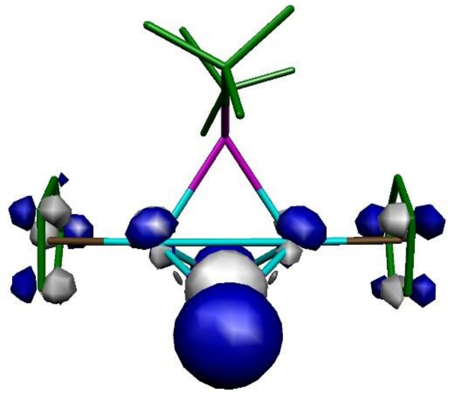
Contributions (%)	Mo´s 13, $P^{t}Bu_2$ 0, P_{ext} 46, P_{cent} 1, Other 40	
MO 108<br><br>σ_{MM} + LP<br><br>-5.72	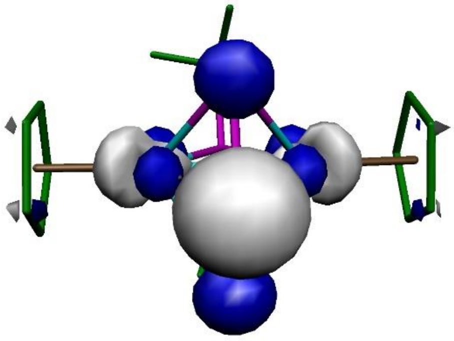	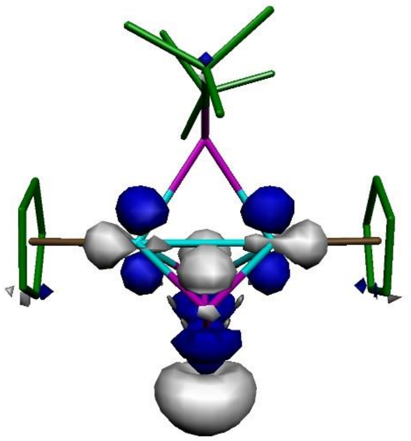
Contributions (%)	Mo´s 53, $P^{t}Bu_2$ 0, P_{ext} 18, P_{cent} 23, Other 7	

MO 107<br>$\sigma_{M2P\mathit{t}Bu2}$<br>-5.87	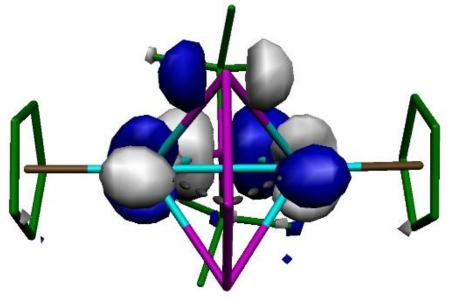	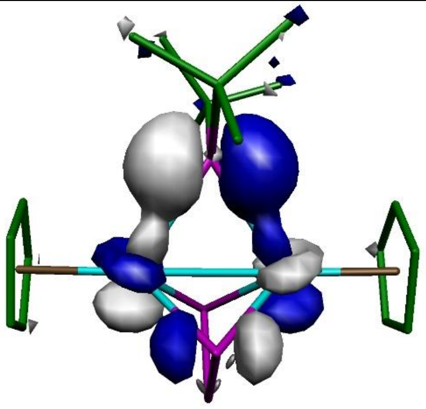
Contributions (%)	Mo´s 46, P^{t}Bu$_2$ 34, P$_{ext}$ 11, P$_{cent}$ 0, Other 9	
MO 106<br>$\sigma_{M2Pext2}$<br>-6.26	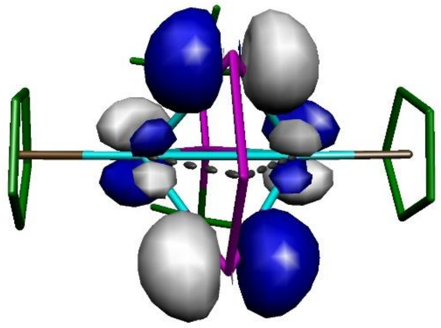	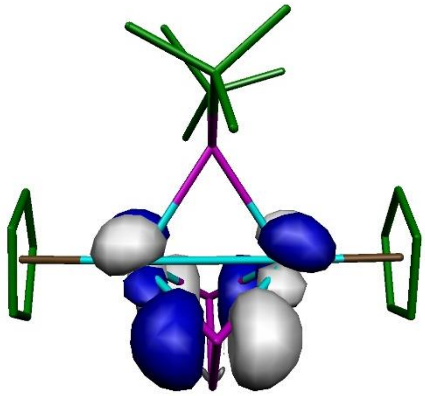
Contributions (%)	Mo´s 49, P^{t}Bu$_2$ 1, P$_{ext}$ 47, P$_{cent}$ 1, Other 3	
MO 102<br>σ_{PP}<br>-7.06	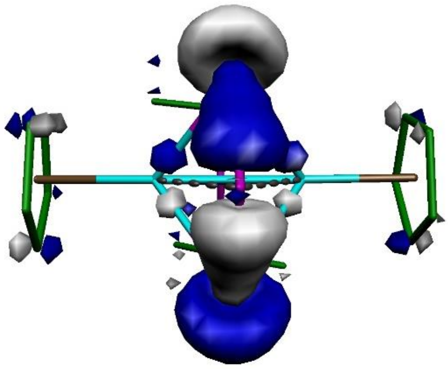	
Contributions (%)	Mo´s 7, P^{t}Bu$_2$ 3, P$_{ext}$ 39, P$_{cent}$ 15, Other 35	

MO 99<br><br>π_{PPP}<br><br>-7.59	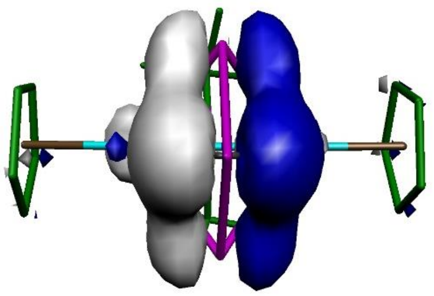	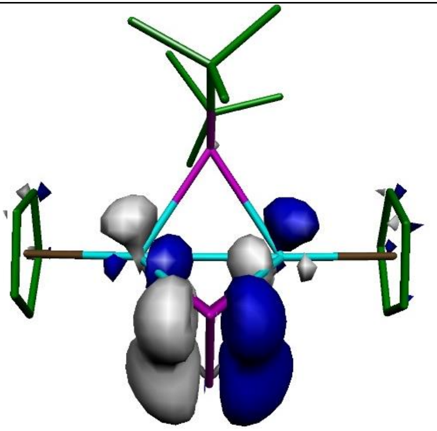
Contributions (%)	Mo´s 38, P^{t}Bu$_2$ 2, P$_{ext}$ 25, P$_{cent}$ 27, Other 7	
MO 98<br><br>$\sigma_{PP} + \pi_{MM}$<br><br>-7.90	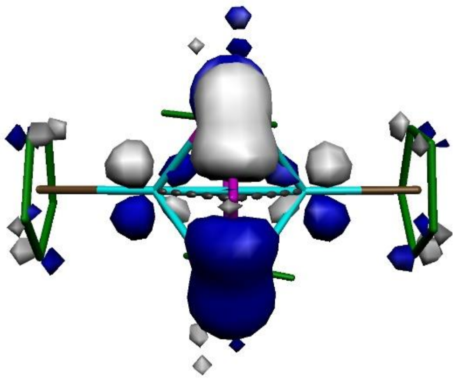	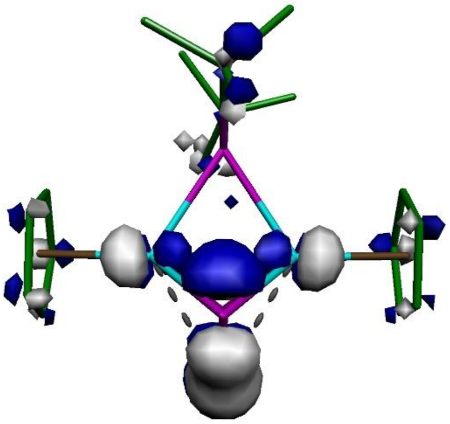
Contributions (%)	Mo´s 35, P^{t}Bu$_2$ 2, P$_{ext}$ 23, P$_{cent}$ 13, Other 27	

Table S2: M06L-DFT computed topological properties of the electron density at the Bond Critical Points in complex **2** (labels according to xyz file) and some simple P_n species [a]

Bond	ρ	$\nabla^2(\rho)$	ε
	compound ***2***		
Mo1 – Mo2	0.516	1.262	0.200
Mo1 – P50	0.569	2.241	0.231
Mo1 – P51	0.575	2.258	0.253
Mo2 – P50	0.572	2.234	0.229
Mo2 – P51	0.574	2.245	0.253
Mo1 – P52	0.379	2.369	2.237
Mo2 – P52	0.377	2.378	2.599
Mo1 – P^tBu_2	0.554	2.968	0.101
Mo2 – P^tBu_2	0.554	2.928	0.100
P50 – P52	0.722	-1.861	0.020
P51 – P52	0.728	-1.906	0.005
	Free P_3^- (d_{PP} = 2.068 Å; P–P–P = 71.75°)		
P1 – P3	0.882	-4.070	0.114
P2 – P3	0.882	-4.069	0.114
	P_3^-/forced angle (d_{PP} = 2.062 Å; P-P-P = 107.35°)		
P1 – P3	0.911	-4.535	0.220
P2 – P3	0.912	-4.549	0.220
	P_4 (d_{PP} = 2.185 Å)		
P – P	0.750	-2.279	0.051
	P_4(B3LYP) (d_{PP} = 2.218 Å)		
P – P	0.715	-1.998	0.048

[a] Values of the electron density at the bond critical points (ρ) are given in eÅ^{-3}; values of the laplacian of ρ at these points ($\nabla^2\rho$) are given in eÅ^{-5}.

Figure S4. M06L-DFT computed molecular orbitals of P_3^- with fixed P–P–P angle at 107.35°, viewed from a point close to the P_3 plane (left), with their energies (in eV) indicated below). On the right, a view of these orbitals from a plane perpendicular to the above one.

View 1	View 2
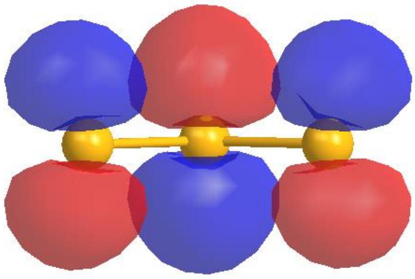	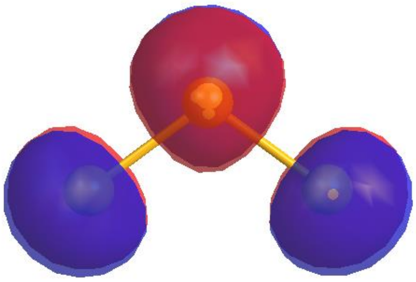
LUMO+1 (+2.36 eV)	
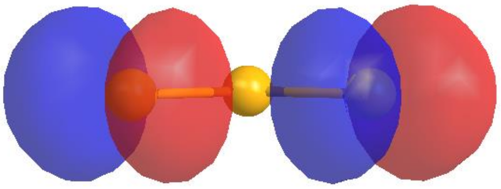	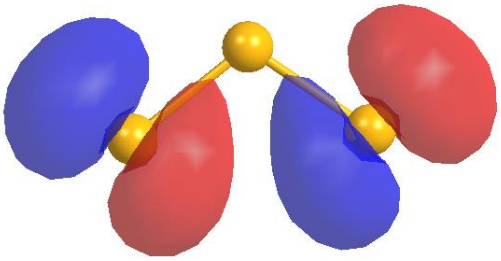
LUMO (+0.61 eV)	
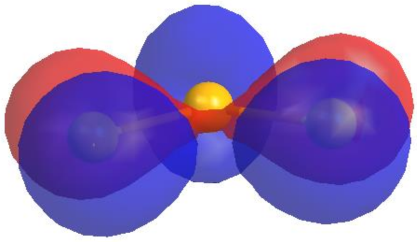	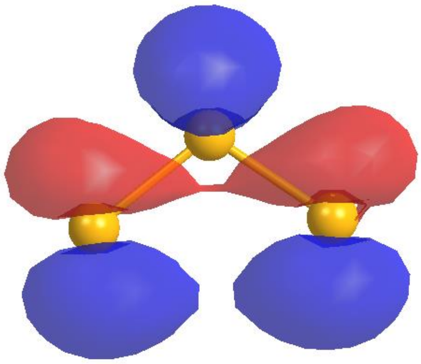
HOMO (+0.49eV)	

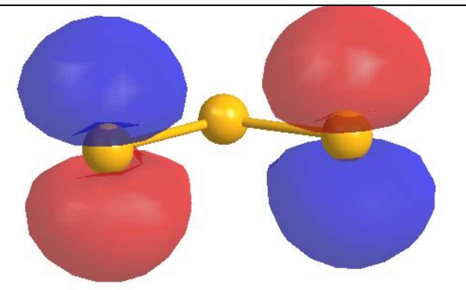
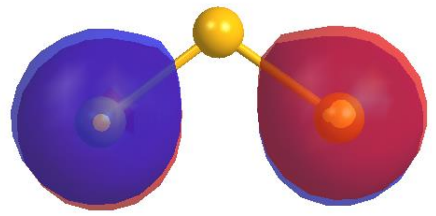

HOMO-1 (+0.14eV)

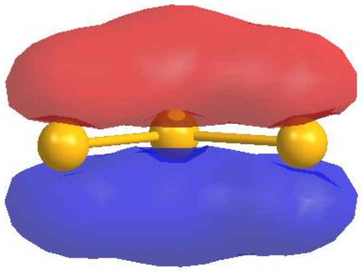
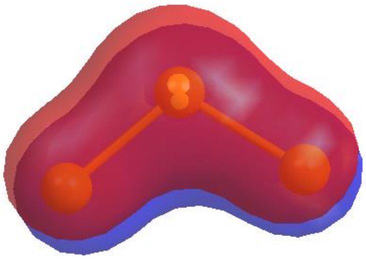

HOMO-2 (-2.20 eV)

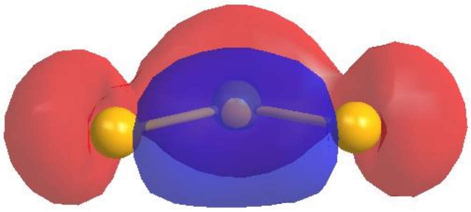
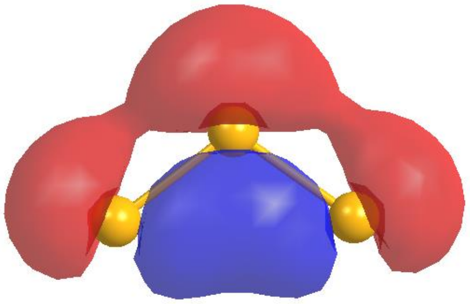

HOMO-3 (-2.43eV)

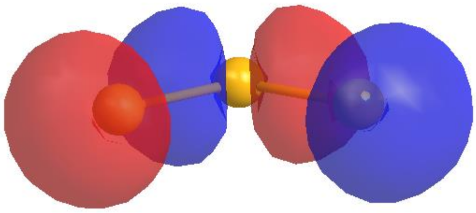
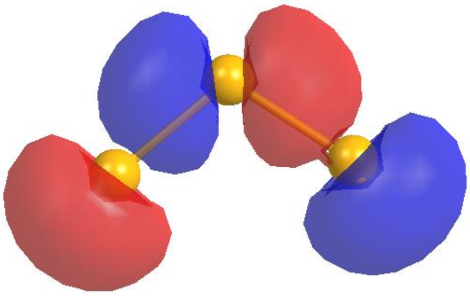

HOMO-4 (-2.52eV)

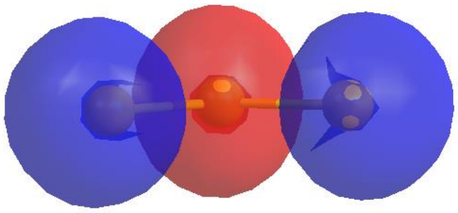	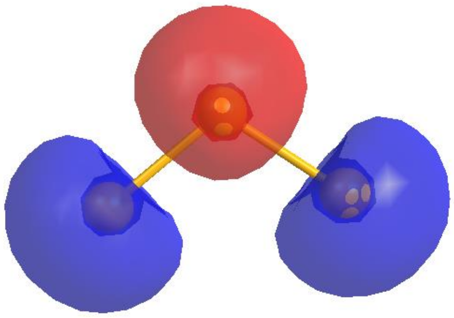
HOMO-5 (-6.04eV)	
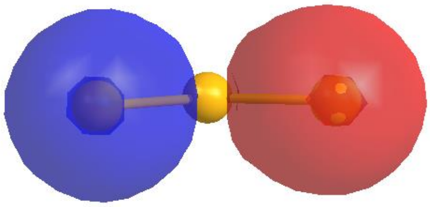	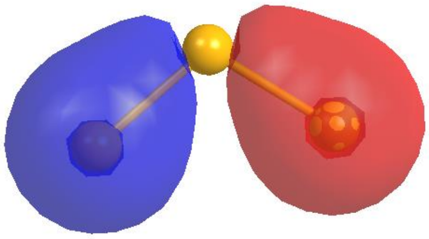
HOMO-6 (-9.41eV)	
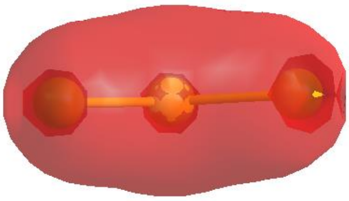	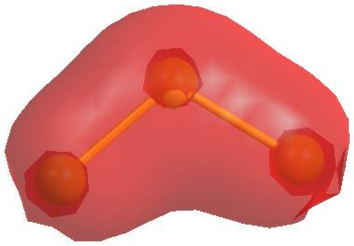
HOMO-7 (-12.48eV)	

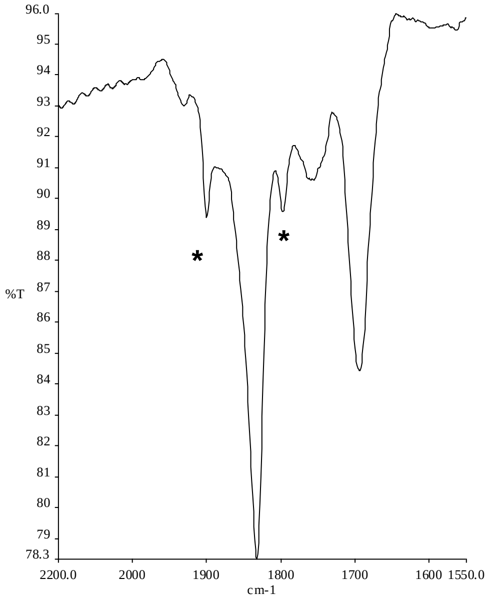

Figure S5. IR spectrum of compound **5** in tetrahydrofuran solution. Bands marked (*) correspond to a trace of Na[MoCp(CO)$_3$] present in the reaction mixture.

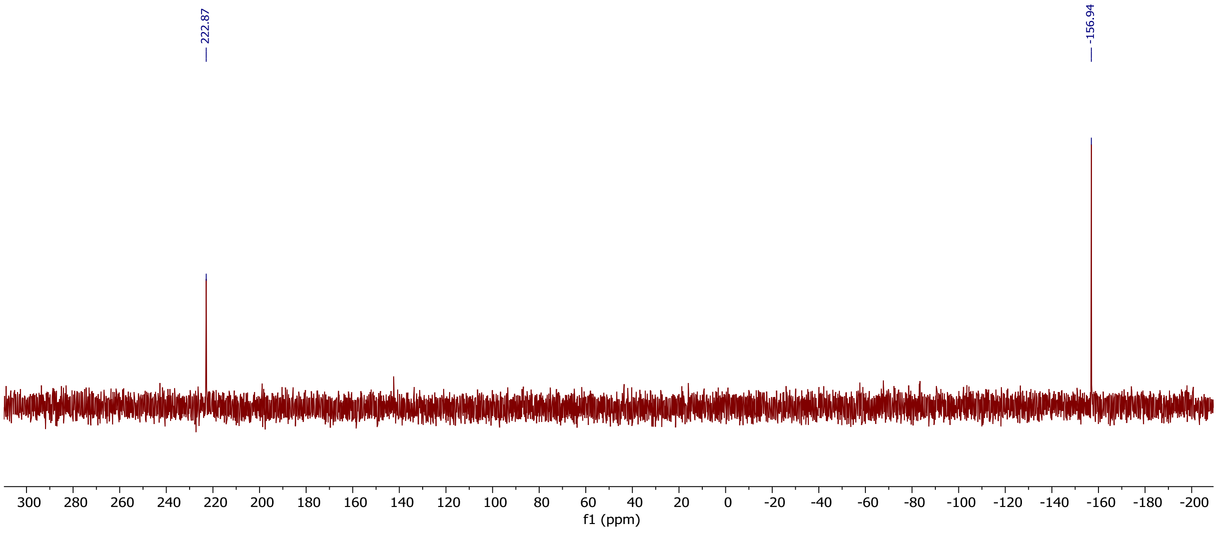

Figure S6. $^{31}P\{^1H\}$ NMR spectrum of compound **5** in tetrahydrofuran solution.

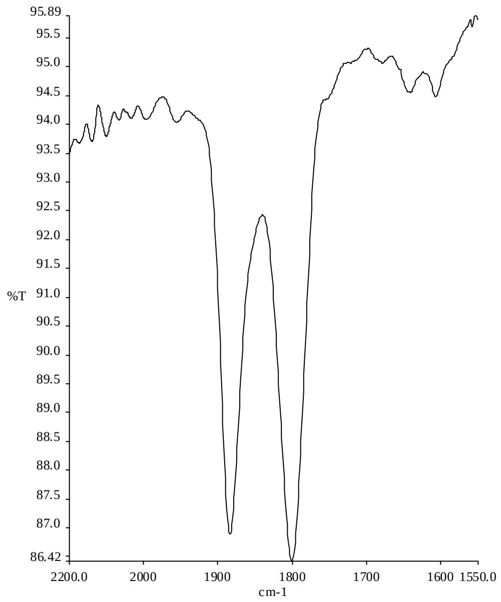

Figure S7. IR spectrum of compound **3** in dichloromethane solution.

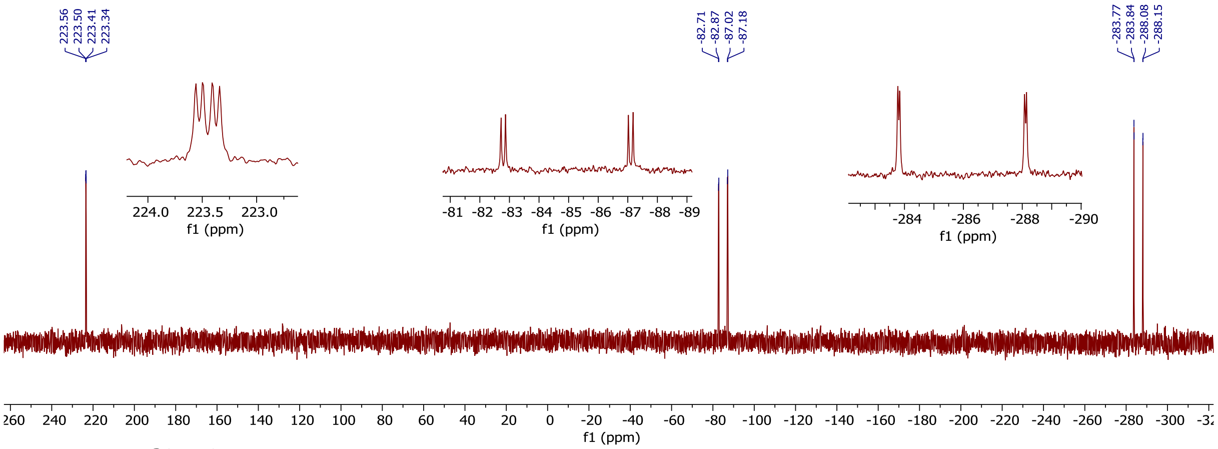

Figure S8. $^{31}P\{^1H\}$ NMR spectrum of compound **3** (C_6D_6).

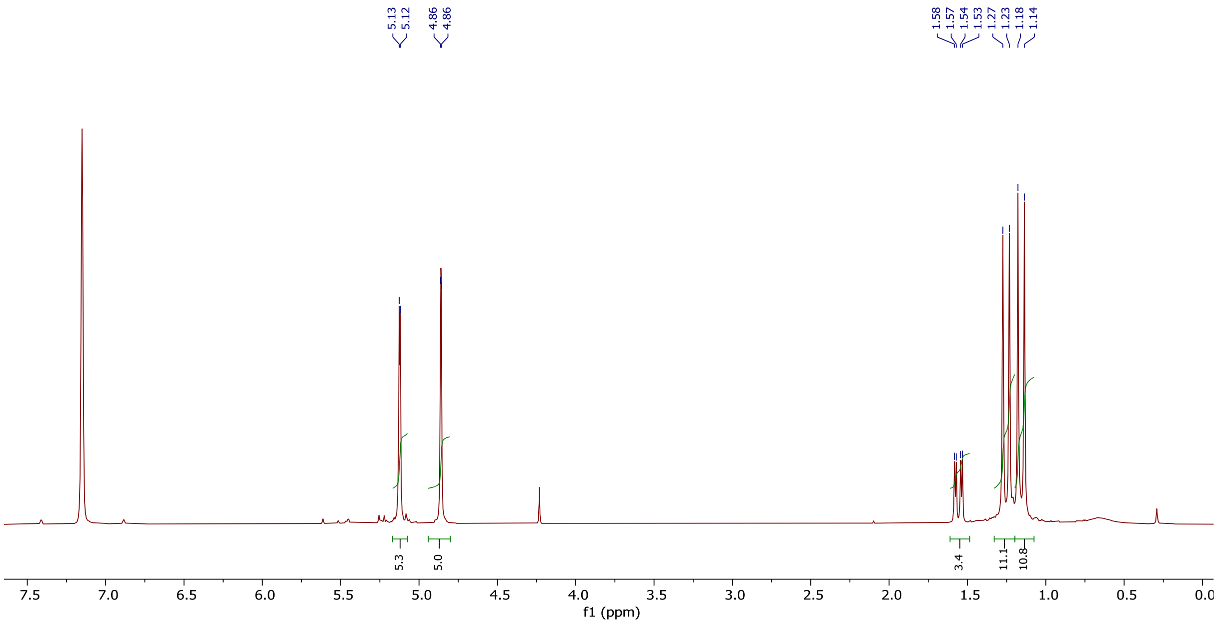

Figure S9. ¹H NMR spectrum of compound **3** (C_6D_6).

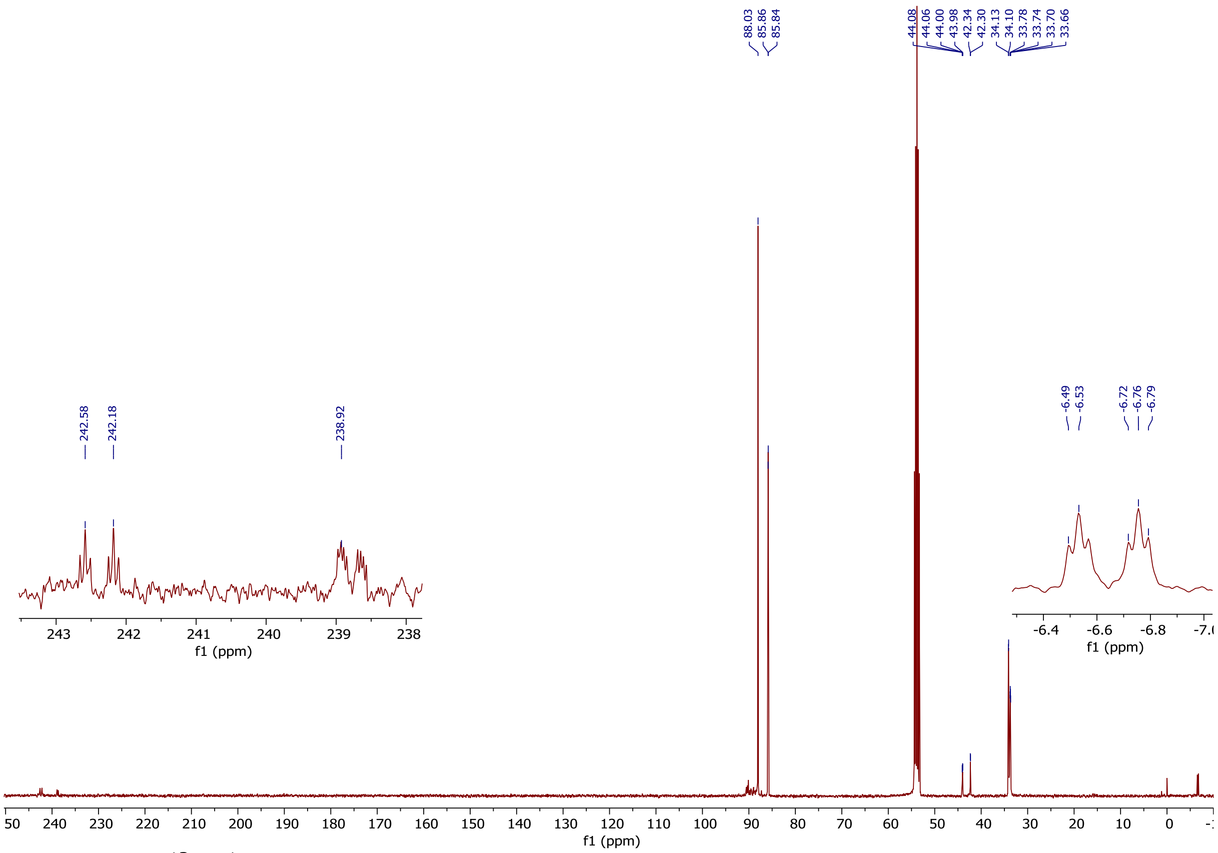

Figure S10. ¹³C{¹H} NMR spectrum of compound **3** (CD_2Cl_2).

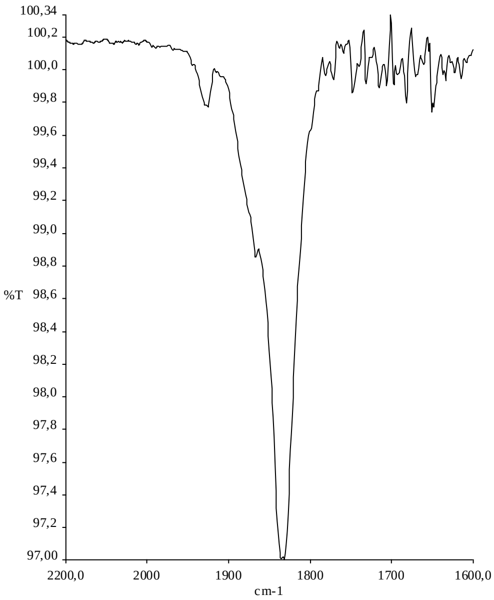

Figure S11. IR spectrum of compound **6** in dichloromethane solution.

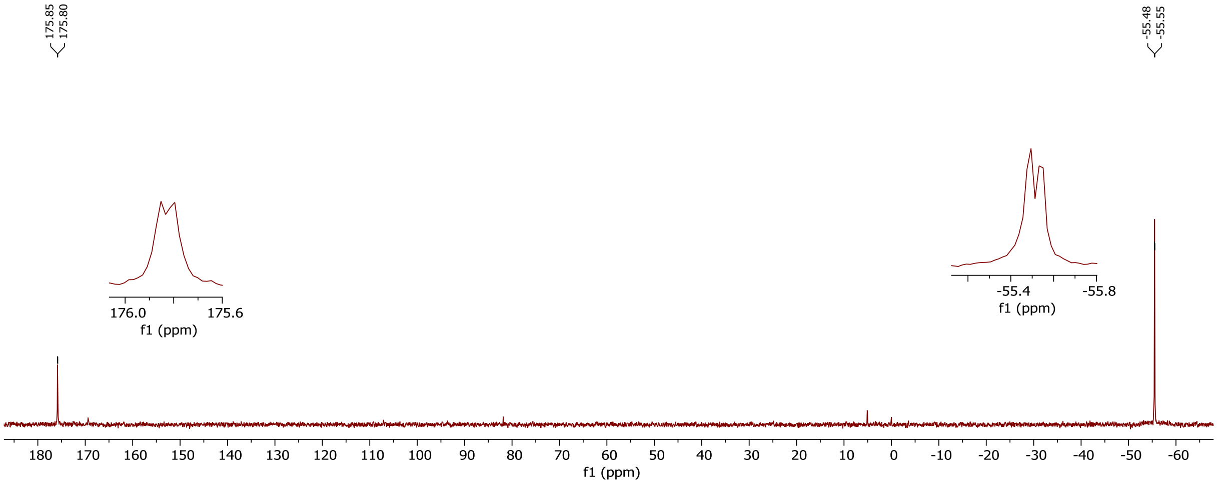

Figure S12. $^{31}P\{^{1}H\}$ NMR spectrum of compound **6** (C_6D_6).

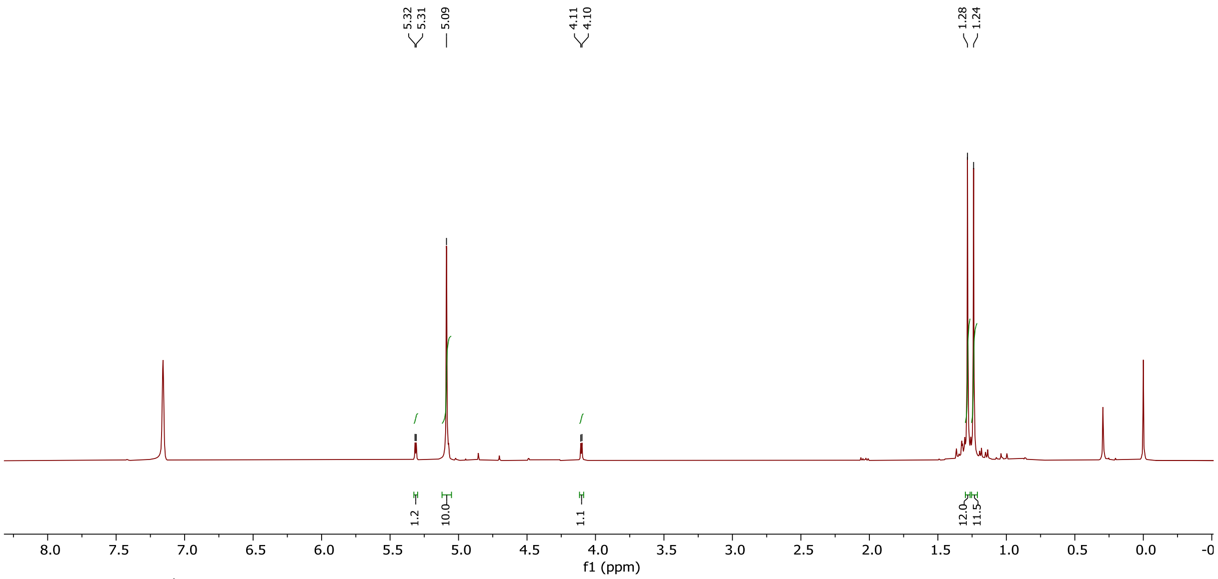

Figure S13. ^{1}H NMR spectrum of compound **6** (C_6D_6).

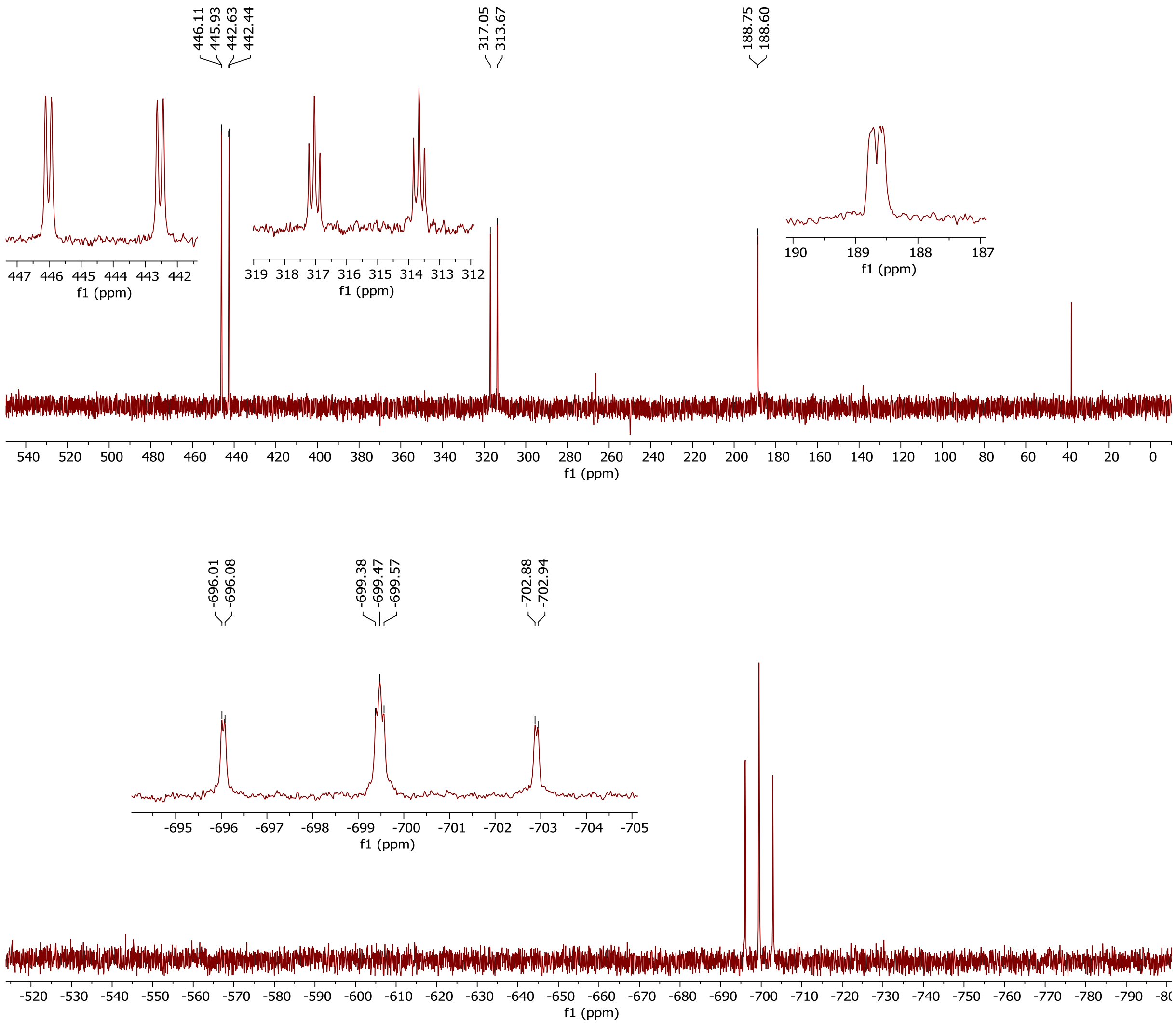

Figure S14. $^{31}P\{^{1}H\}$ NMR spectra of compound **7** (low-field and high-field regions, CD_2Cl_2).

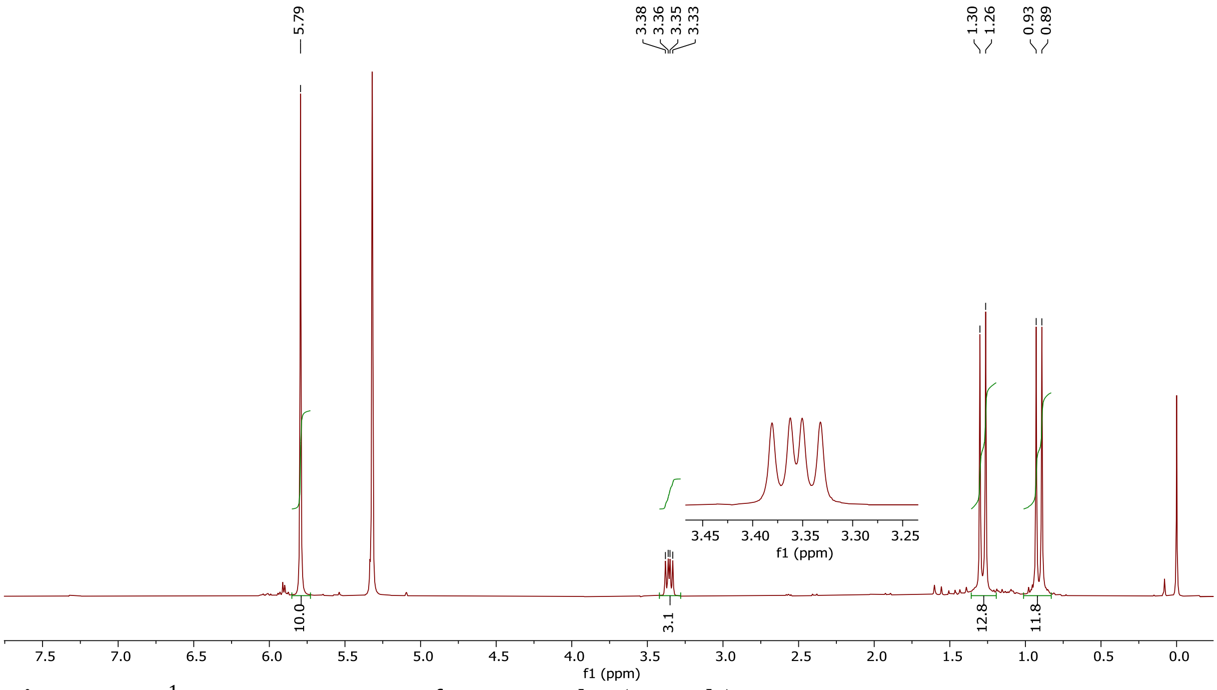

Figure S15. ^{1}H NMR spectrum of compound **7** (CD_2Cl_2).

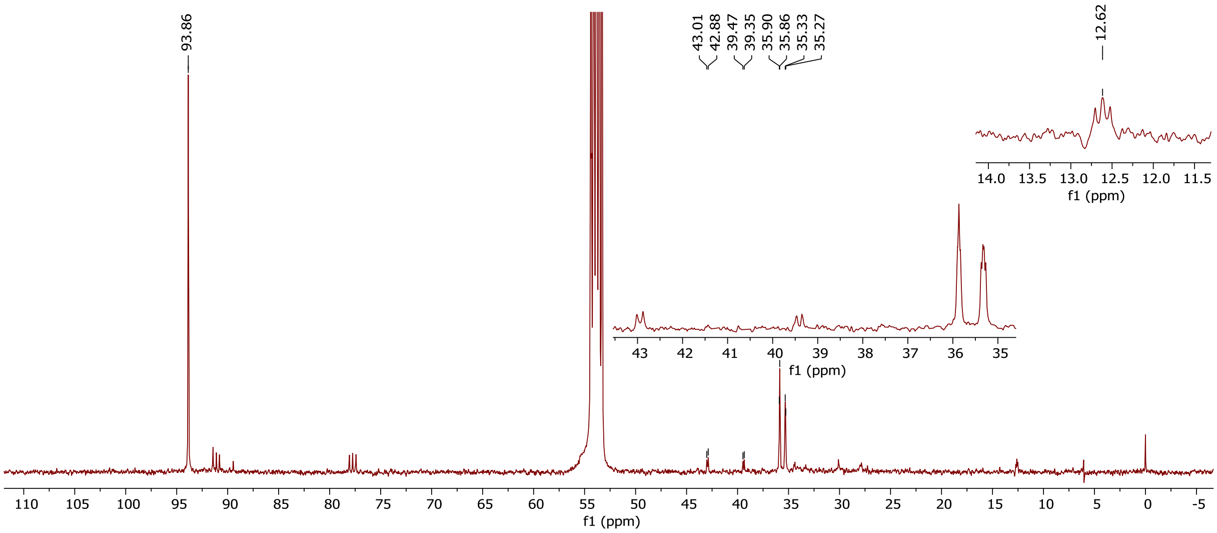

Figure S16. $^{13}C\{^{1}H\}$ NMR spectrum of compound **7** (CD_2Cl_2).

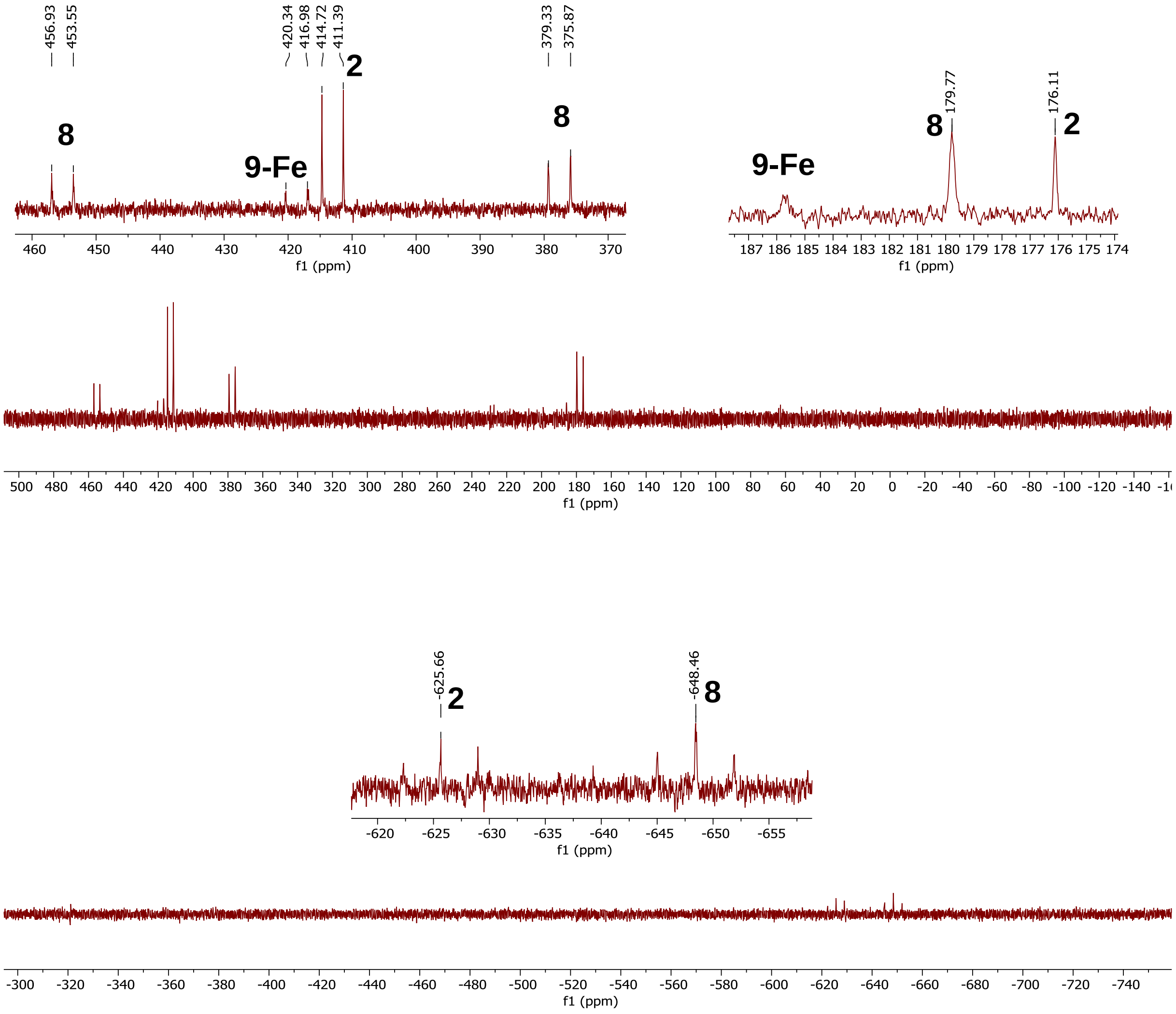

Figure S17. $^{31}P\{^1H\}$ NMR spectra of compound **8** (low-field and high-field regions, toluene), impurified with the parent compound **2** and with some **9-Fe**.

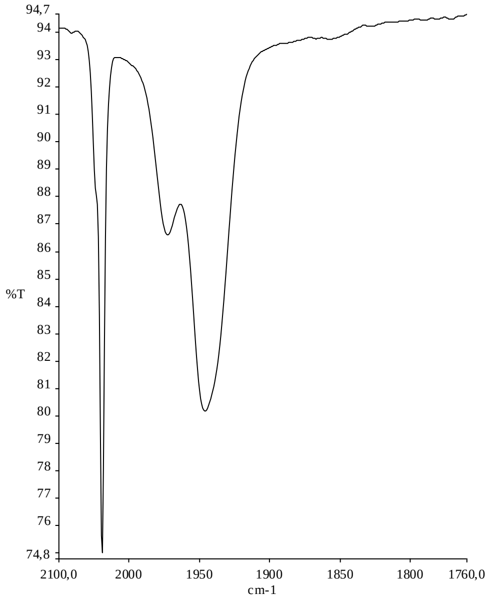

Figure S18. IR spectrum of compound **9-Fe** in dichloromethane solution.

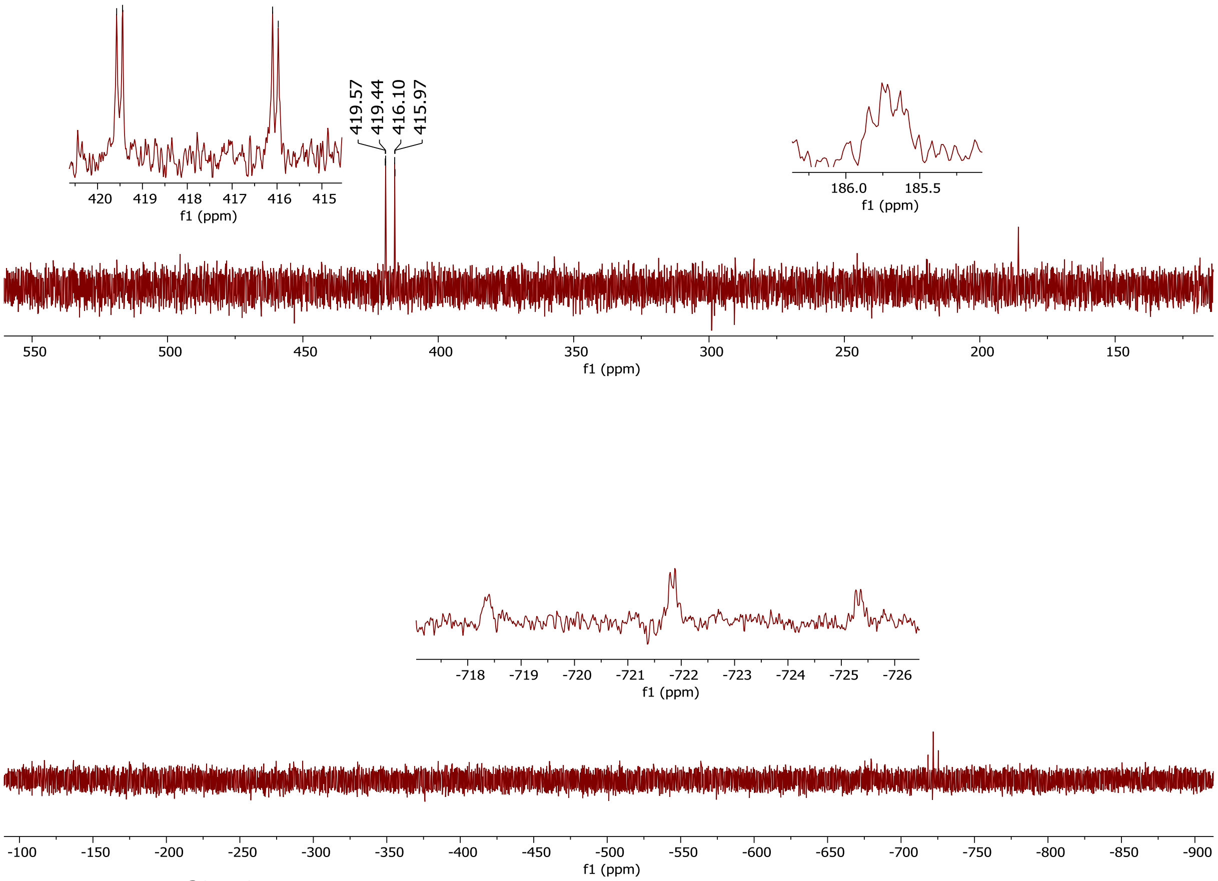

Figure S19. $^{31}P\{^{1}H\}$ NMR spectra of compound **9-Fe** (low-field and high-field regions, C_6D_6),

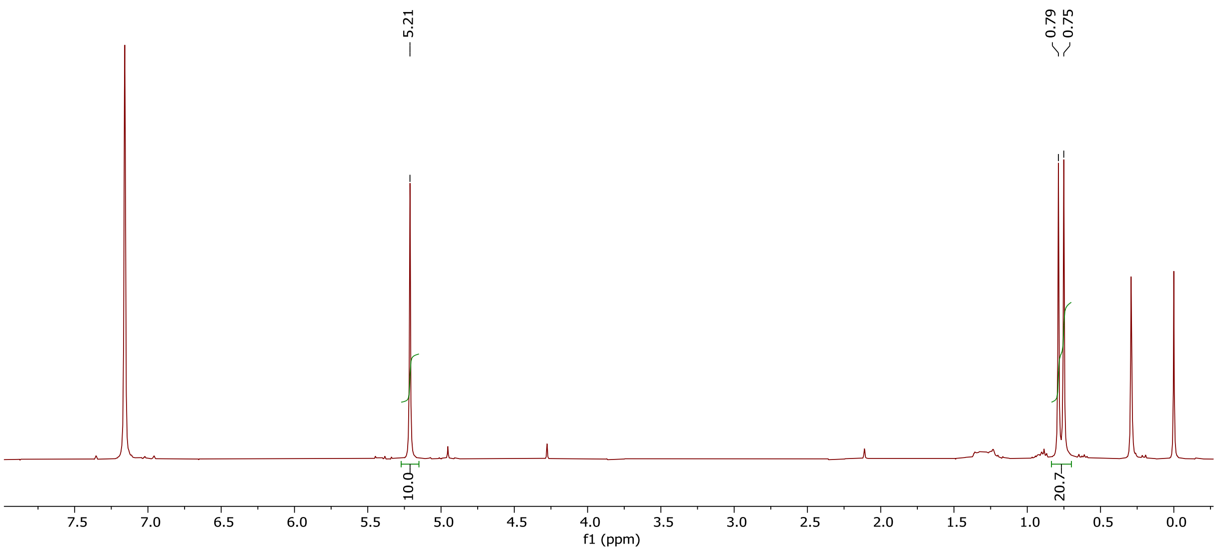

Figure S20. ^{1}H NMR spectrum of compound **9-Fe** (C_6D_6).

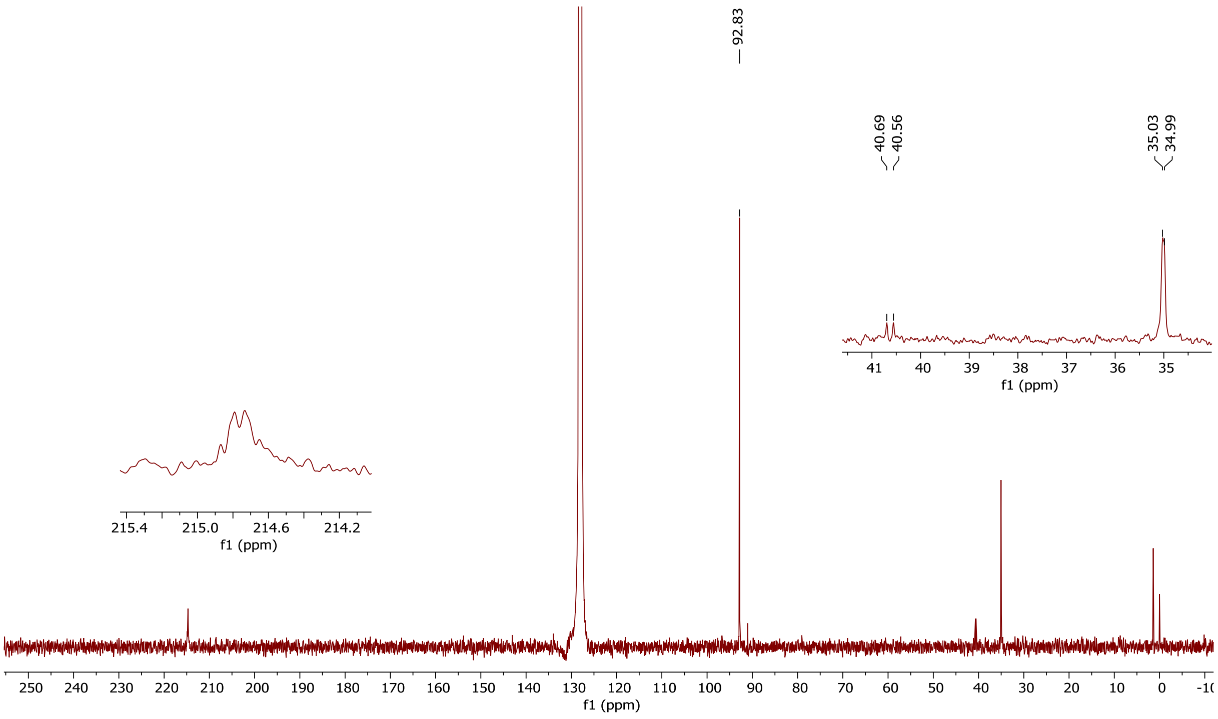

Figure S21. $^{13}C\{^{1}H\}$ NMR spectrum of compound **9-Fe** (C_6D_6).

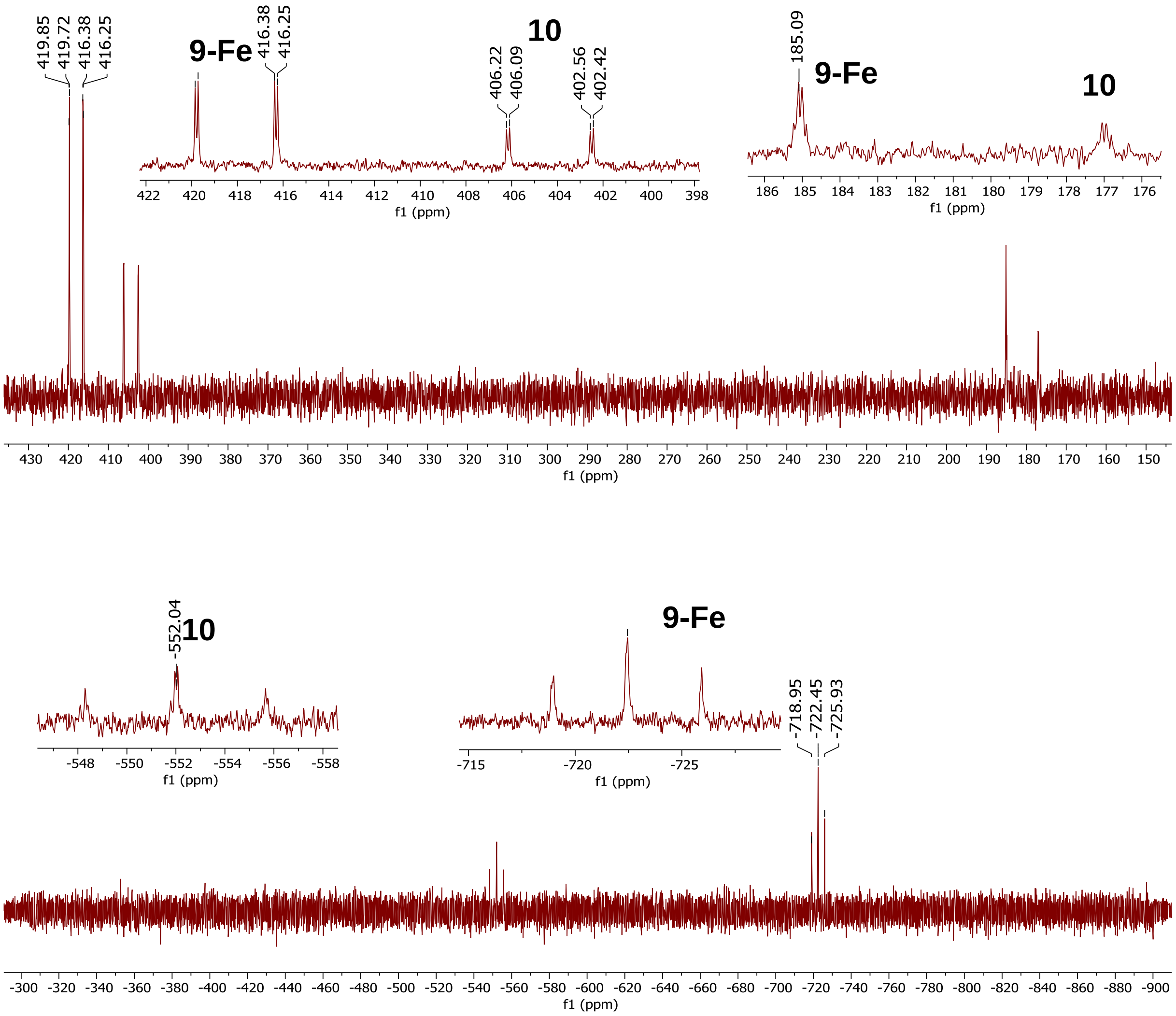

Figure S22. $^{31}P\{^{1}H\}$ NMR spectra of a mixture of compounds **9-Fe** and **10** (low-field and high-field regions, toluene)

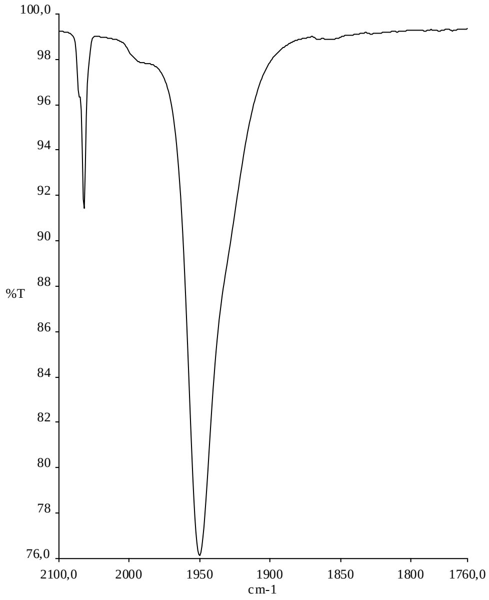

Figure S23. IR spectrum of compound **9-Mo** in dichloromethane solution.

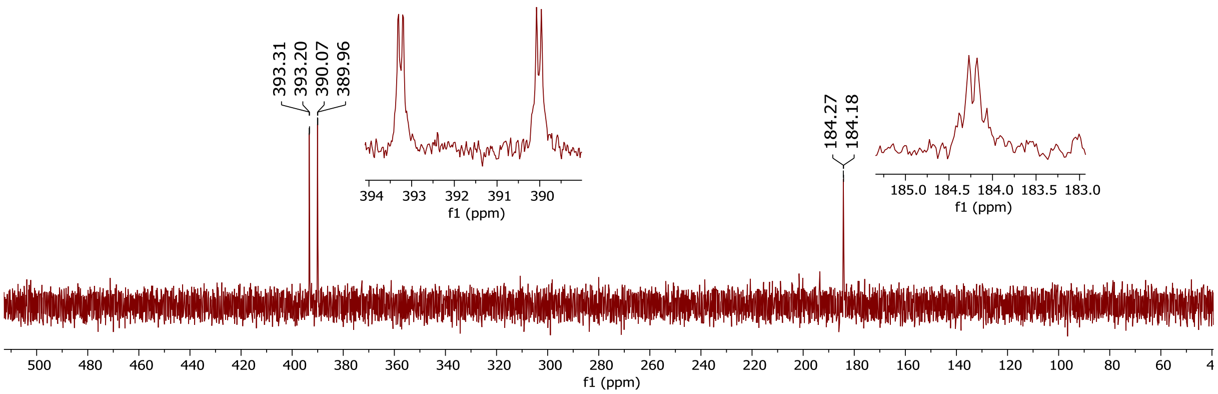

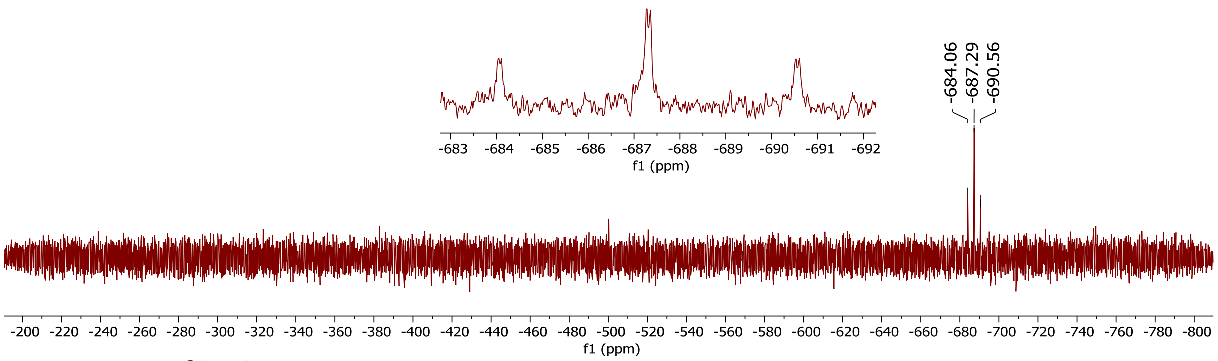

Figure S24. $^{31}P\{^{1}H\}$ NMR spectra of compound **9-Mo** (low-field and high-field regions, CD_2Cl_2).

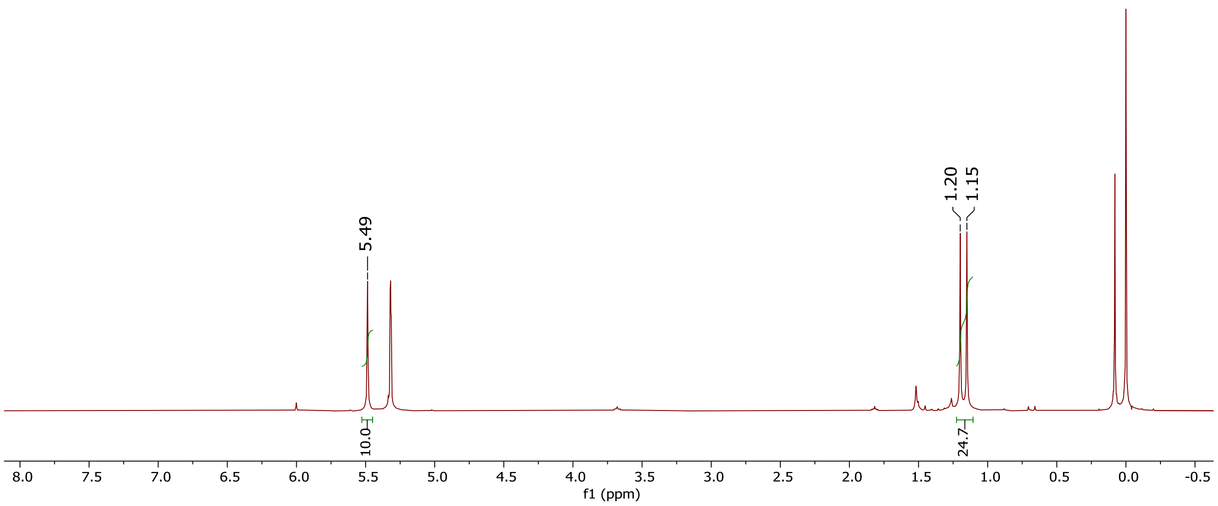

Figure S25. ^{1}H NMR spectrum of compound **9-Mo** (CD_2Cl_2).

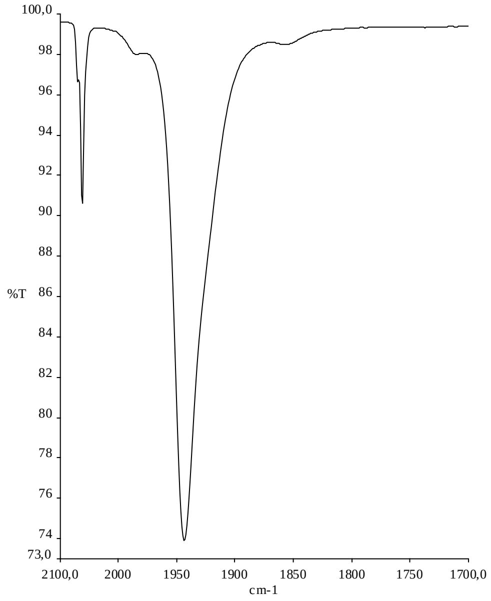

Figure S26. IR spectrum of compound **9-W** in dichoromethane solution.

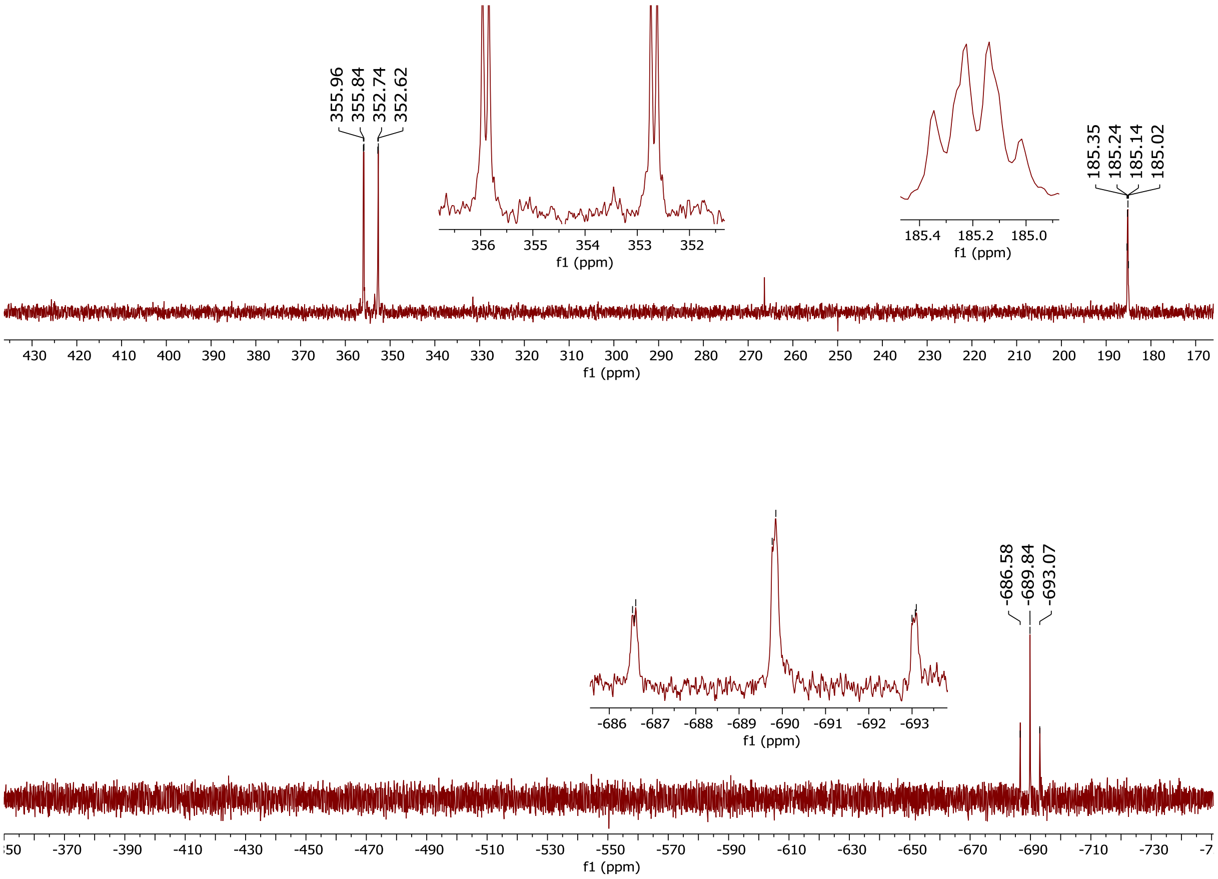

Figure S27. $^{31}P\{^{1}H\}$ NMR spectra of compound **9-W** (low-field and high-field regions, CD_2Cl_2)

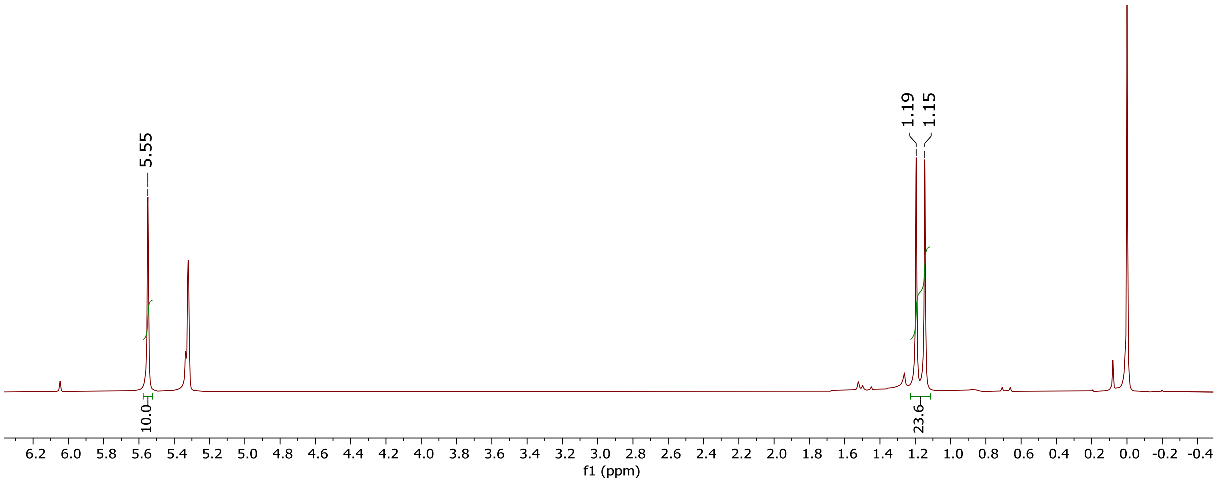

Figure S28. ^{1}H NMR spectrum of compound **9-W** (CD_2Cl_2)

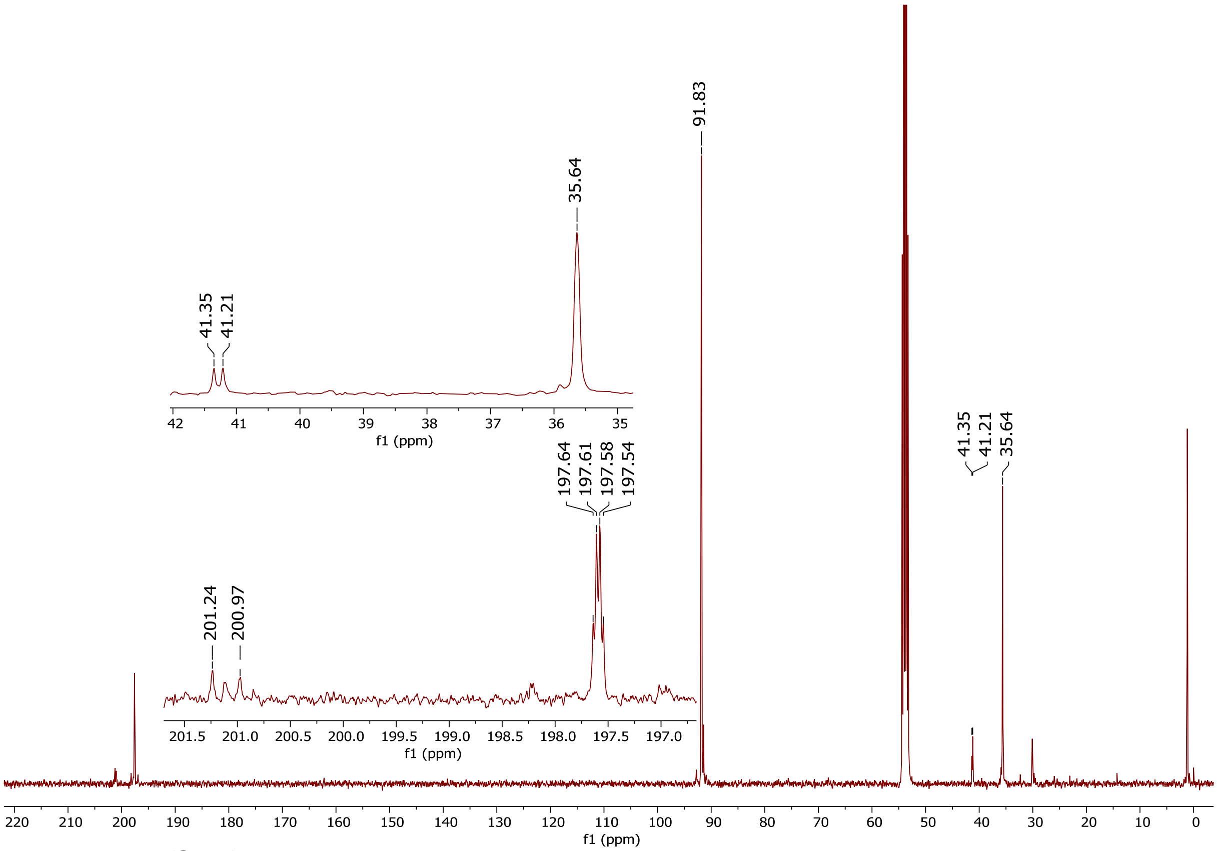

Figure S29. $^{13}C\{^{1}H\}$ NMR spectrum of compound **9-W** (CD_2Cl_2)

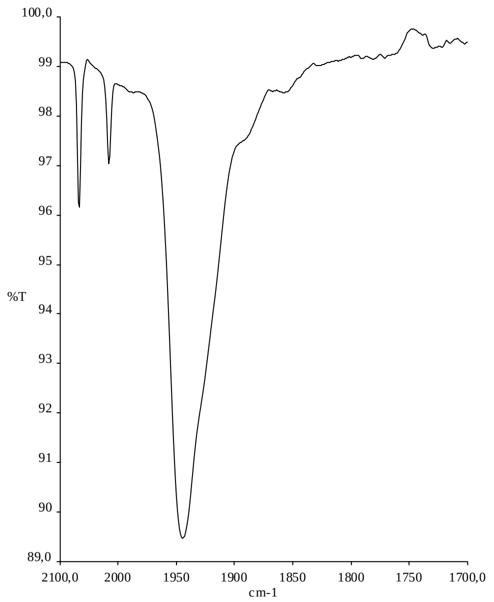

Figure S30. IR spectrum of compound **11** in dichoromethane solution.

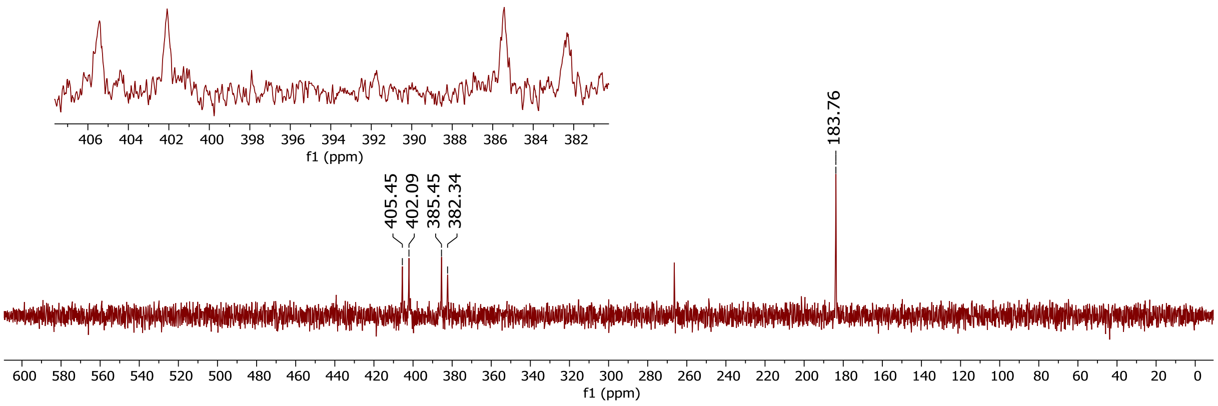

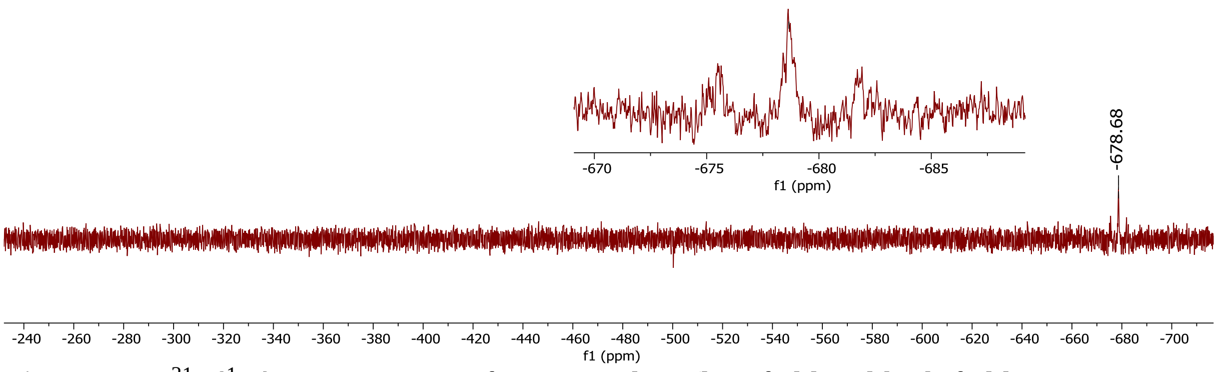

Figure S31. $^{31}P\{^{1}H\}$ NMR spectra of compound **11** (low-field and high-field regions, CD_2Cl_2)

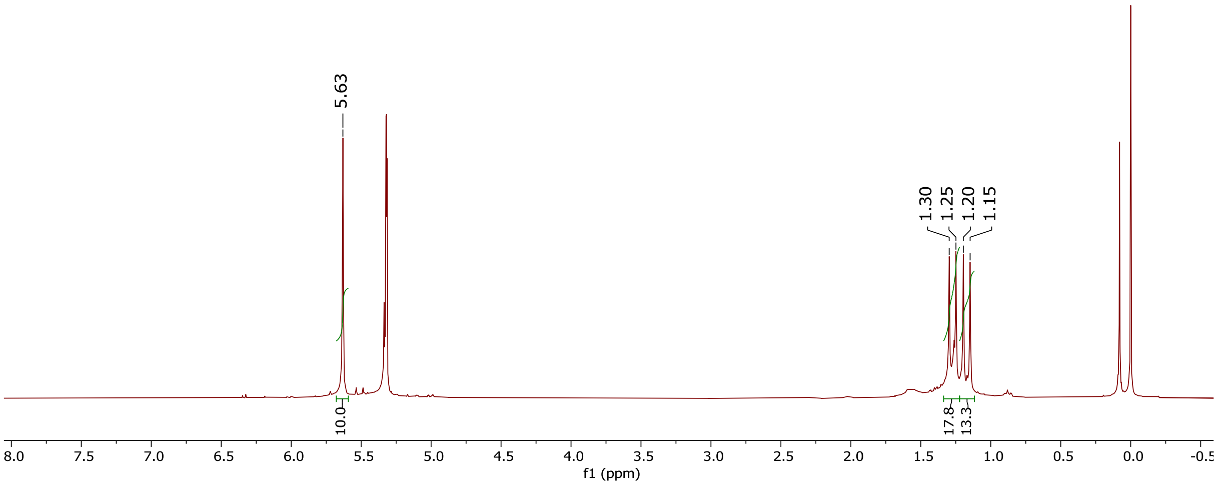

Figure S32. ^{1}H NMR spectrum of compound **11** (CD_2Cl_2).
